# Discovering New Tyrosinase Inhibitors by Using In Silico Modelling, Molecular Docking, and Molecular Dynamics

**DOI:** 10.3390/ph18030418

**Published:** 2025-03-16

**Authors:** Kevin A. OréMaldonado, Sebastián A. Cuesta, José R. Mora, Marcos A. Loroño, José L. Paz

**Affiliations:** 1 Departamento Académico de Química Fisicoquímica, Facultad de Química e Ingeniería Química, Universidad Nacional Mayor de San Marcos, Lima 15081, Peru; kevin.ore1@unmsm.edu.pe; 2 Grupo de Química Computacional y Teórica (QCT-USFQ), Departamento de Ingeniería Química, Universidad San Francisco de Quito, Diego de Robles y Vía Interoceánica, Quito 170901, Ecuador; sebastian.cuesta@postgrad.manchester.ac.uk; 3 Department of Chemistry, Manchester Institute of Biotechnology, The University of Manchester, Manchester M17DN, UK; 4 Departamento Académico de Química Inorgánica, Facultad de Química e Ingeniería Química, Universidad Nacional Mayor de San Marcos, Lima 15081, Peru; jpazr@unmsm.edu.pe

**Keywords:** tyrosinase, melanoma, QSAR, molecular docking, molecular dynamics

## Abstract

**Background/Objectives:** This study was used in silico modelling to search for potential tyrosinase protein inhibitors from a database of different core structures for IC_50_ prediction. **Methods**: Four machine learning algorithms and topographical descriptors were tested for model construction. **Results**: A model based on multiple linear regression was the most robust, with only six descriptors, and validated by the Tropsha test with statistical parameters R^2^ = 0.8687, Q^2^_LOO_ = 0.8030, and Q^2^_ext_ = 0.9151. From the screening of FDA-approved drugs and natural products, the pIC_50_ values for 15,424 structures were calculated. The applicability domain analysis covered 100% of the external dataset and 71.22% and 73.26% of the two screening datasets. Fifteen candidates with pIC_50_ above 7.6 were identified, with five structures proposed as potential tyrosinase enzyme inhibitors, which underwent ADME analysis. **Conclusions**: The molecular docking analysis was performed for the dataset used in the training-test process and for the fifteen structures from the screening dataset with potential pharmaceutical tyrosinase inhibition, followed by molecular dynamics studies for the top five candidates with the highest predicted pIC_50_ values. The new use of these five candidates in tyrosinase inhibition is highlighted based on their promising application in melanoma treatment.

## 1. Introduction

Tyrosinase is a type of dinuclear copper ion oxidoreductase that acts as a rate-limiting enzyme, catalysing two distinct reactions in melanin biosynthesis: the hydroxylation of monophenols to o-diphenols and the oxidation of o-diphenols to the corresponding o-quinones [1]. It plays a key role in processes such as pigmentation in vertebrates and the browning of foods [2]. Activation of tyrosinase is therefore closely linked to melanogenesis [3], the process by which melanin is synthesised. In short, melanin is produced by the oxidation and polymerisation of tyrosinase and plays a vital role in the normal functioning of bio-organisms [4,5,6]. However, excessive melanin production leads to some pigmentation disorders, such as melanoma, melasma, freckles, and post-inflammatory hyperpigmentation. Melanoma is a form of skin cancer caused by the excessive proliferation of melanocytes [7,8].

Tyrosinase has therefore been considered an important target for the development of therapeutic agents for pigmentation disorders and the treatment of melanoma. Researchers have increasingly focused on tyrosinase inhibitors to prevent or treat such disorders [9]. In addition, tyrosinase expression and its action within the microenvironment are closely associated with neurodegeneration [10], Parkinson’s disease [11,12], and Alzheimer’s disease [13,14]. Therefore, the development of a safe and effective tyrosinase inhibitor is considered highly relevant for the scientific community.

In recent years, several experimental (in vitro) and computational (in silico) studies have been conducted to identify structures with tyrosinase enzyme inhibitory activity. For instance, Sepehri et al. [15] evaluated the inhibitory activity of twelve 3-hydroxy-4-pyridinones (3,4-HPOs), revealing that some of the tested compounds demonstrated superior inhibitory activity compared to kojic acid, a well-established inhibitor commonly used as a positive control in enzymatic inhibition assays. The investigation was complemented by a molecular docking study on both human and fungal enzymes (PDB ID 2Y9X). On the other hand, Amaral et al. [16] designed and synthesised a series of thioxo-dihydroquinazolinone compounds, and the protein–ligand interactions were evaluated by molecular docking. The authors also performed computer-assisted pharmacokinetic studies, which provided valuable information on these new potential tyrosinase inhibitors for drug development, including their absorption, distribution, and dosage optimisation. Other studies have investigated specific groups of compounds with inhibitory activity, such as flavonoids, which are common polyphenols found in fruits, vegetables, and beverages [17,18]. Additionally, purely computational (in silico) studies have evaluated the inhibitory activity of structures from various chemical classes known for their inhibitory properties, such as phenols, terpenes, steroids, chalcones, flavonoids, alkaloids, and long-chain fatty acids, originating from synthetic, semi-synthetic, or natural sources [19].

The aim of this study was centred on modelling based on the quantitative structure–activity relationship (QSAR) approach to obtain models based on 2D and 3D topographic descriptors. The dataset was composed of organic compounds, including analogs of cinnamyl esters with cinnamic acid (CA) compounds and paeonol, compounds obtained from Vinca major L. extract and its secondary metabolites, N-(4-methoxyphenyl) caffeamide-induced compounds, compounds based on 3-heteroarylcoumarins, derivatives of cinnamic acid esters, flavones, substituted vanillyl cinnamate analogues, quercetin, and rifampicin [20,21,22,23,24,25,26,27,28], which are reported as inhibitors of the fungal tyrosinase enzyme (Agaricus bisporus, PDB ID 2Y9X). The best and most robust model was used for virtual screening of FDA-approved drugs (DrugBank 5.0) [29] and structures of natural products MEGx—Isolated Natural Products of microbial or plant origin [30]. Additionally, the protein–ligand interaction of tyrosinase from Agaricus bisporus (2Y9X) was evaluated through molecular docking and molecular dynamics studies.

## 2. Results and Discussion

### 2.1. Selection of Variables

**Figure 1:**
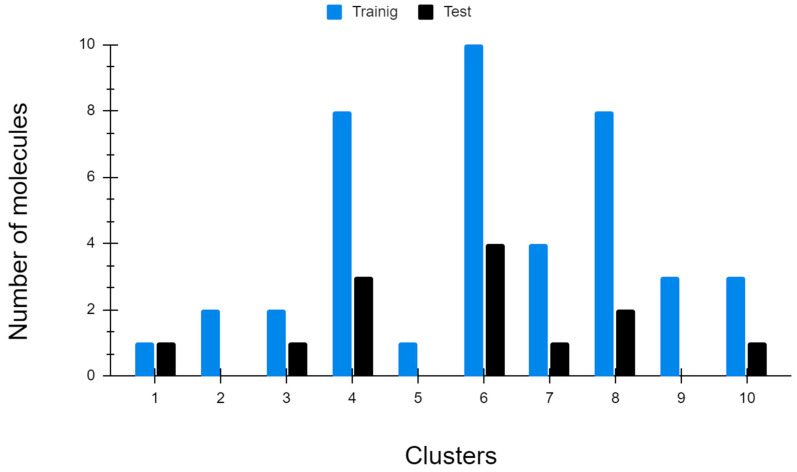
The dataset was divided into 10 groups using Ward’s method to divide it into blue bars (training 74%) and black bars (test set 26%).

The dataset, comprising compounds with pIC_50_ values ranging from 3.22 to 7.52, was used for modelling (Table S1 and Figure S1). This range allows for the evaluation of a diverse set of bioactive compounds. From the model selection process, 103 models were generated (see Supporting Information, Table S2), and the top 10, based on the best statistical parameters, were selected for further evaluation. In this second phase, descriptors were filtered to optimise the modelling process. The data was split into 75% training and 25% test sets to assess the predictive power of the models [31], using Ward’s cluster analysis method (Figure 1). Finally, models 3, 4, 6, and 9 (see Table 1) were selected to determine the best-performing model.

### 2.2. Model Performance and Validation

For the case of models selected with the multiple linear regression approach, the QSAR-INSUBRIA (QSARINS) software v224 [31,32,33,34] was used to validate the models. In QSARINS it is possible to sort the calculated models, displaying all or only a selected part of the best models, according to several criteria: fit (highest R^2^), robustness (highest Q^2^_LOO_), stability (lowest R^2^ − Q^2^_LOO_), lowest correlation between descriptors and highest correlation with response (low K_XX_ and high K), and root mean square errors in training computation (RMSE_TR_), training prediction by leave one out (LOO) (RMSE_CV_), and external prediction set (RMSE_EXT_). The most stable, predictive, and generalisable model should have the least difference between fit, CV, and external parameters and, consequently, RMSE values as similar as possible [32]. The models were validated both internally and externally, again using Tropsha’s criteria [33] as shown in Table 2, selecting the model with the best benchmark R^2^, Q^2^_CV_, Q^2^_ext_, and RMSE values. Finally, four models that passed the validation tests met the evaluation criteria. The selected models are presented in Table 1, and the best model was model 3, obtained with Multiple Linear Regression (MLR), which met the evaluation criteria with Q^2^_ext_ greater than 0.915, Q^2^_LOO_ greater than 0.803, and similar RMSE values.

The molecular descriptors and Equation (1) were obtained through the model M3. Among the descriptors selected from this model, linear index descriptors—derived from mapping and linear function principles within geometry-based matrices—were identified as the most robust for predicting bioactivity values in this context, surpassing the predictive capabilities of other descriptor types. These topographic descriptors capture structural information not typically encoded by alternative descriptor families, offering unique insights into molecular topology. Furthermore, they exhibit variability that is comparable to or even surpasses that of descriptors generated by widely used molecular descriptor calculation software, as supported by prior studies [35,36,37]. Additionally, the descriptors were computed using physicochemical properties that provide insights into the compound’s intrinsic nature, as they provide insights into its molecular size, shape, electronic distribution, and potential for intermolecular interactions. Of the six descriptors from the 3M model, five are classified as 3D indices (GV [3], TS [6], TS [5], GV [6], and ES), and one as a 2D index (HM). These indices reflect specific physicochemical properties: electronegativity (e), polarizability (p), topological polar surface area (PSA), refractivity (r), and Van der Waals volume (v) (see Table S3).

M3: pIC_50_ = 0.0138GV3 − 0.3314TS6 + 0.0763HM − 0.6033TS5 − 0.1315GV6 + 2.0047ES + 3.3397 (1)

### 2.3. Virtual Screening

The applicability domain was evaluated using the ranges, Euclidean distance to mean, city block distance to mean, and probability density methods. The analysis showed 100% coverage for the external data structures and the structures obtained from the screening with higher pIC_50_, demonstrating the robustness of the model and the reliability of the predictions.

Model 3M was applied to an external database obtained from DrugBank, which includes FDA-approved drug molecules, as well as a set of natural product molecules from Analyticon Discovery [38]. The pIC_50_ values of these compounds were predicted to identify potential new drug candidates for tyrosinase inhibition, which could be useful in melanoma treatment. The top 15 compounds, with predicted pIC_50_ values ranging from 7.534 to 8.357, were selected. Additionally, applicability domain analysis was performed on the screening dataset, resulting in 71.22% coverage for the DrugBank data and 73.26% coverage for the natural product data from Analyticon Discovery. All 15 selected compounds met the established criteria for the applicability domain (see Tables S4 and S5).

### 2.4. Absorption, Distribution, Metabolism, and Excretion (ADME) Predictions

SwissADME uses five freely available prediction models: XLOGP3 [39], WLOGP [40], MLOGP [41], SILICOS-IT [42], and iLOGP [43]. The consensus log Po/w is the arithmetic mean of the values predicted by the five proposed methods. In Table S6, we can see that the log Po/w values obtained from the 15 promising compounds are found with values below 3.7. We selected the first five compounds with the most favourable pIC_50_ values and evaluated their lipophilicity and water solubility.

**Figure 2:**
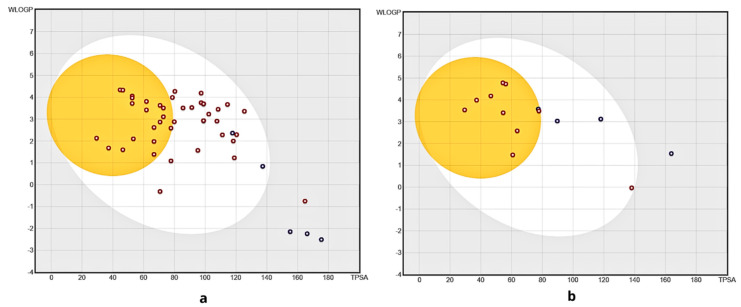
BOILED−Egg diagram for the model data set (**a**), and for the selected compounds from the screening data predicted (**b**).

Figure 2 shows the BOILED-Egg diagram of the model data and the best compounds from the external data, used for the evaluation of passive gastrointestinal absorption (HIA) and blood–brain barrier (BBB), based on the physicochemical descriptors WLOGP and TPSA, for lipophilicity and apparent polarity [44]. It is observed that the model data (Figure 2a) have an intermediate bioavailability, with a higher distribution of compounds in the gastrointestinal absorption (GIA) region, in terms of absorption or blood–brain barrier (BBB) penetration. And four compounds (12, 14, 15, and 18 in Table S1, in the Supplementary Material) remained in the grey region, indicating poor intestinal absorption and poor ability to cross the blood–brain barrier.

**Figure 3:**
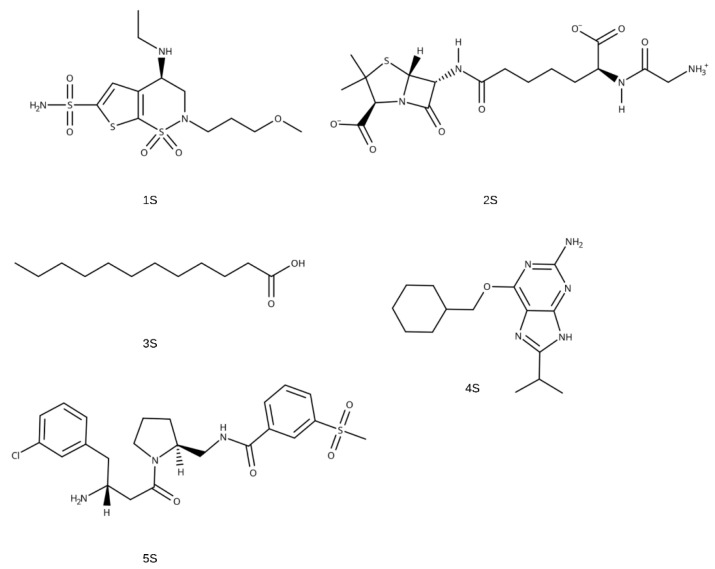
Two−dimensional structures of the five best compounds obtained in the screening.

**Figure 4:**
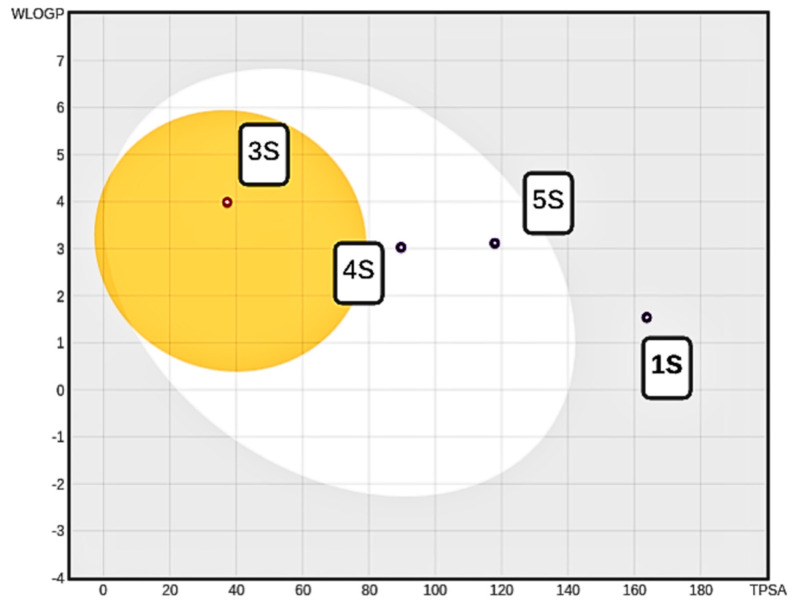
BOILED−Egg diagram for compounds 1S, 3S, 4S, and 5S.

The ADME analysis for the 15 best candidates identified from the screening data is shown in Figure 2b. One compound falls within the grey region, indicating poor intestinal absorption and limited blood–brain barrier penetration. For a more detailed evaluation of drug potential, the top five compounds, presented in Figure 3, with pIC_50_ values greater than 7.82 (Table 3), were selected. A BOILED-Egg plot analysis was then performed (Figure 4), revealing that four of these five compounds (1S, 3S, 4S, and 5S) have a LogP consensus value below 3.6 (Table S6), suggesting a low lipophilicity and making them promising candidates for drug development.

Compound 1S is the compound that remained in the grey area of the graph. However, it showed a consensus of 0.34, indicating a certain tendency towards neutrality in terms of affinity between the lipid and aqueous phases. This could be suitable for certain applications as it does not show an extreme tendency towards hydrophilicity or lipophilicity. It is reported that compound 1S or brinzolamide, considered a small molecule and approved by the FDA for the treatment of elevated intraocular pressure in patients with ocular hypertension or open-angle glaucoma, acts as a carbonic anhydrase inhibitor used to reduce elevated intraocular pressure in patients [45,46,47]. Compounds 4S and 5S are found in the clear zone, indicating good gastrointestinal absorption. Compound 4S belongs to the class of organic compounds known as hypoxanthines. These are compounds containing the purine derivative 1H-purin-6(9H)-one. It is at an experimental stage and has been linked to the inhibition of CDK2 [48], a protein involved in cell division. Drugs that inhibit CDK2 and prevent cell cycle progression can reduce the sensitivity of the epithelium to many antitumor agents and thus represent a strategy for the prevention of chemotherapy-induced alopecia [49]. On the other hand, compound 5S belongs to the class of organic compounds known as beta-amino acids and derivatives. These are amino acids with a group (-NH2) attached to the beta-carbon atom. It is related to the inhibition of human dipeptidyl peptidase IV (CD26), which has recently attracted considerable interest as a therapeutic approach for the treatment of type 2 diabetes [50]. Compound 3S, known as lauric acid, belongs to the class of organic compounds known as medium-chain fatty acids. It is an inexpensive, non-toxic, and easy-to-handle compound used in laboratory research [51]. It is a rare component of triglycerides and is found in coconut milk, coconut oil, laurel oil, and palm kernel oil. It is also found in breast milk [52]. It has been linked to the induction of apoptosis in cancer and stimulates the proliferation of normal cells by maintaining cellular redox homeostasis [53]. In our studies, it can cross the blood–brain barrier and is absorbed from the intestine with a consensus log Po/w of 3.51, which is consistent with data from ADME studies for this compound reported by DrugBank.

Finally, compound 2S is outside the graph, with a negative value of −2.39, indicating its high hydrophilicity. This compound belongs to the class of organic compounds known as dipeptides. These are organic compounds containing a sequence of exactly two alpha-amino acids linked by a peptide bond [54]. It has been called the “perfect” penicillin and proposed as a potent antimicrobial drug due to its interaction with DD-peptidases, the main clinical defence against bacterial infections [55].

### 2.5. Molecular Docking

The molecular docking studies performed with AutoDock4, both for screening and for internal datasets, provided detailed information on the binding modes of the ligands in the orthosteric site of tyrosinase, as well as the types of interactions involved in the ligand–protein complexes. The docking score of the natural ligand, tropolone, along with the docking scores of the ligands from the internal dataset used to build the QSAR model, were taken as reference values. Additionally, the types of interactions present in the ligand–enzyme complexes (Tables S7 and S8 in the Supplementary Material) were analysed and compared with human tyrosinase (PDB ID: 5M8T).

It is worth noting that tropolone, along with kojic acid (included in the modelling data), is commonly used as a reference in molecular docking studies due to its inhibitory activity against tyrosinase. In this context, we focused our study on fungal tyrosinase (PDB ID: 2Y9X), as experimental data support its inhibition and its use for in silico studies [20,21,22,23,24,25,26,27,28]. Moreover, this enzyme presents a structural similarity in its inhibition site with human tyrosinase (PDB ID: 5M8T), whose crystalline structure also includes tropolone as its natural ligand, with an RMSD of 0.048 Å compared to tropolone bound to fungal tyrosinase (Figure S2 and Table S7).

Tropolone interactions primarily involve histidine residues, specifically HisA259 and HisA263. Notably, carbon–hydrogen interactions occur with HisA259, while pi-pi stacking interactions are observed with HisA263, which are characteristic of systems containing aromatic rings. Additionally, tropolone forms a pi-sigma interaction with ValA283 [6] (see Figure S2).

The interaction modes and binding affinities of the five selected compounds from the molecular docking study are summarised in Figures S3 and S4 and Table 4. These summaries include details on hydrogen bond interactions, interaction distances, docking score (D.S.) values, and predicted pIC_50_ values.

Interestingly, all five ligands interacted with Phe 264, while additional interactions were observed with Asn 260, Val 283, His 263, His 85, and His 259. These findings emphasise the critical role of His A259 and His A263 in both the reference ligand (tropolone) and the proposed ligands, highlighting them as key residues of interest. The proposed ligands docked within the same active site as tropolone but exhibited higher D.S. values compared to the natural ligand, suggesting potentially stronger binding affinities.

The docking scores of 15 compounds from the screening dataset were evaluated through triplicate calculations to obtain average docking score (D.S. Average) values. Compounds 1S to 5S exhibited favourable negative D.S. values at the active site, ranging from −4.577 to −8.950 kcal/mol (see Table 5). Their predicted pIC_50_ values were also calculated, and compounds 1S to 5S, which had the highest pIC_50_ values and favourable D.S. values, were selected for further analysis (see Figures S2 and S3 and Table 6).

Additionally, the conformation with the most negative docking score from the triplicate analyses was considered the most likely binding conformation and was used as the starting structure for the molecular dynamic simulations (Figure S4).

### 2.6. Molecular Dynamics

The structural stability of the ligand–receptor complexes over 200 ns of molecular dynamics (MD) simulations was evaluated using root mean square deviation (RMSD) and root mean square fluctuation (RMSF) of residue positions. These parameters allowed us to characterise both the persistence of ligand binding and the ligand-induced flexibility in tyrosinase, providing key insights into interaction modes and conformational stability.

**Figure 5:**
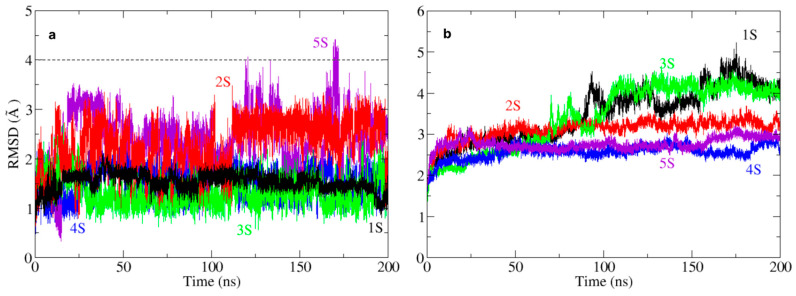
Root mean square deviation (RMSD) of the plots of the screening ligands, compounds 1S to 5S (**a**), and RMSD of the protein (**b**), throughout the 200 ns molecular dynamics (MD) simulation.

The RMSD profiles of the ligands (Figure 5a, Figures S5 and S6) revealed variability in binding stability. Specifically, ligands 1S, 3S, and 4S exhibited relatively low RMSD values (<2.5 Å), indicating stable retention within the active site. In contrast, 2S and 5S showed greater fluctuations, particularly 5S, which exceeded 4 Å at ~170 ns, suggesting lower binding stability. This difference in dynamic behaviour may be related to the nature of the interactions formed, as well as the structural compatibility of each ligand with the active site environment.

Regarding the stability of tyrosinase (Figure 5b), RMSD analysis revealed differences in ligand-induced conformational reorganisation. Tyrosinase remained relatively stable (<3 Å) with ligands 3S and 5S, while complexes with 2S and 4S exhibited moderate fluctuations (3–4 Å). Notably, ligand 1S induced a progressive increase in protein RMSD, surpassing 4 Å by the end of the simulation. This behaviour suggests that 1S may trigger conformational rearrangements of the active site, potentially favouring alternative structural states.

**Figure 6:**
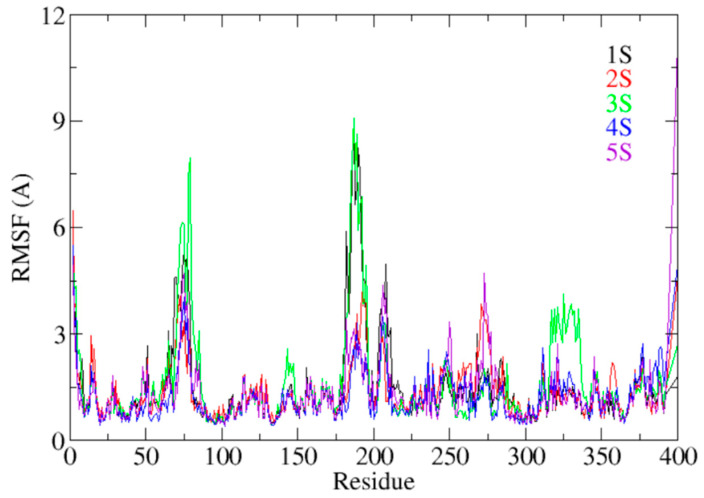
Plots of the RMSFs of the screening ligands during the 200 ns MD simulation.

RMSF analysis (Figure 6 and Figure S7) provided insights into the flexibility of individual residues in response to ligand binding. Residues located in loops and helices adjacent to the active site showed higher fluctuations in the presence of 1S and 2S, which could be linked to their lower binding stability. Conversely, complexes with 3S and 4S displayed lower RMSF variations, suggesting greater conformational restraint imposed by these ligands. In particular, residues H244 and E256, which form key hydrogen bonds with 1S and 2S, exhibited significant fluctuations, indicating transient interactions that may modulate local flexibility within the active site.

From an energetic perspective (Table 6), the complexes exhibited differences in stabilisation mechanisms. Interaction energy analysis revealed that 1S had the highest van der Waals contribution (−127.101 kcal/mol), suggesting significant hydrophobic coupling with the active site, while 3S showed the weakest interaction in this regard (−100.239 kcal/mol). Electrostatic interactions were particularly relevant for 2S (−43.783 kcal/mol), indicating strong ionic or dipolar contributions, whereas 1S and 5S displayed positive values, suggesting some degree of electrostatic repulsion within the active site.

**Figure 7:**
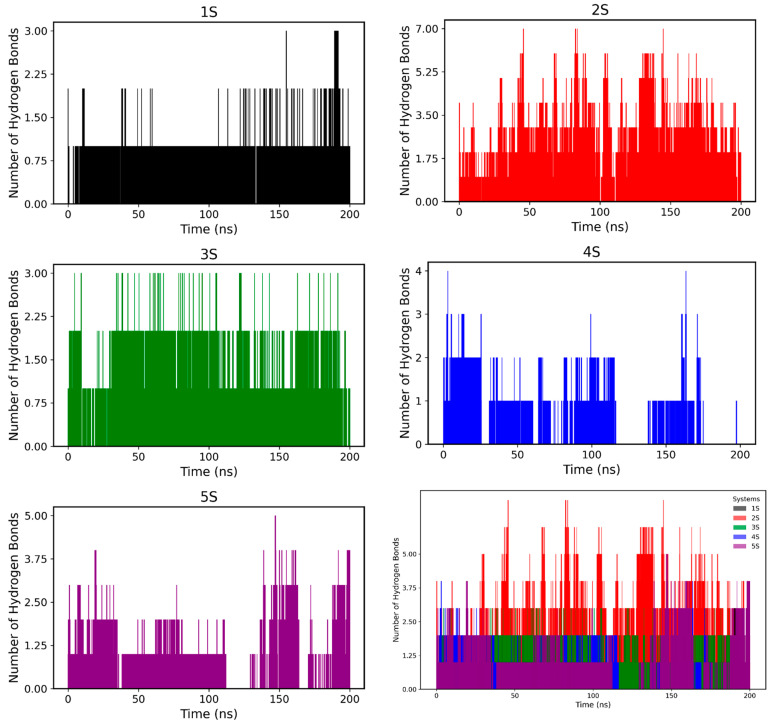
Number of hydrogen bonds between the enzyme tyrosinase and the screening ligands 1S to 5S, investigated as drug candidates over a 200 ns molecular dynamics simulation.

**Figure 8:**
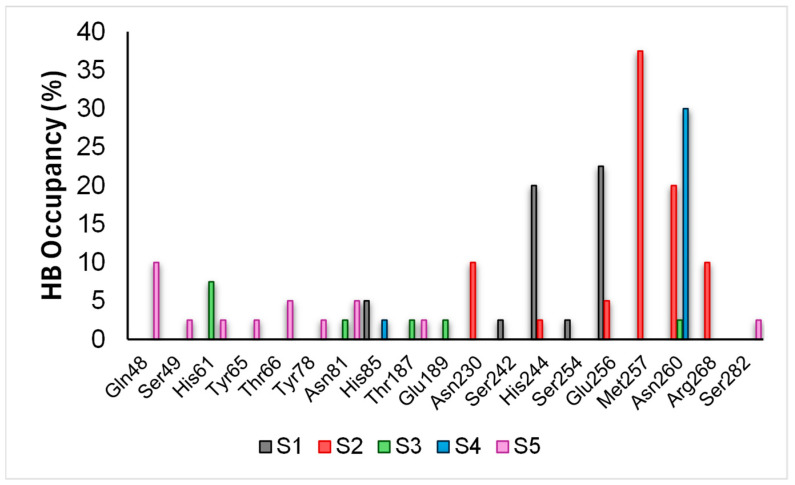
HB over time of compounds 1S to 5S.

Hydrogen bond (HB) analysis (Figure 7 and Figure 8) provided detailed information on ligand binding modes. Ligand 1S formed hydrogen bonds with the flexible loop residue H244 and the α-helix residue E256, with relatively high occupancies (>20%), indicating a significant contribution to its binding mode. However, the dynamic behaviour observed in RMSD and RMSF suggests that these interactions may be transient, facilitating conformational reorganisation of the active site. In the case of 2S, its partial exit from the active site was accompanied by the formation of more stable interactions with peripheral residues such as N260 and M257, which may have contributed to its lower binding stability within the active site.

On the other hand, ligands 3S and 4S exhibited more persistent interactions. Ligand 3S formed hydrogen bonds with H61 and several active site residues with moderate occupancies (<10%), while 4S showed a particularly stable interaction with N260 (30% occupancy). Finally, ligand 5S, due to its extended structure, formed multiple transient interactions with residues such as Q48, S49, and H61, but with generally low occupancies (<10%), explaining its higher RMSD and lower binding stability.

The stability of the complexes and the observed binding modes suggest that ligands such as 4S and 3S may represent better candidates in terms of conformational stability and interaction persistence. Specifically, the low flexibility induced by 4S suggests that its binding may be more effective in blocking the active site. In contrast, ligands such as 2S and 5S, which exhibited higher mobility and lower interaction stability, may require structural modifications to optimise their affinity and retention within the active site. Furthermore, if the flexibility induced by 1S is related to an inhibition mechanism involving active site reconfiguration, its potential as an inhibitor should not be dismissed without further investigation (see Figures S8 and S9 for more details).

## 3. Materials and Methods

### 3.1. Dataset

The work of Tang K et al. [20] was initially used, from which a background review [21,22,23,24,25,26,27,28] was conducted and used to construct a database of 54 molecular structures that reported experimental IC50 values. From the review, those structures that reported inhibitory activity using kojic acid as a reference were selected to undergo further filtering and standardisation of IC50 values. It is worth noting that this approach was successfully used by us in a previous article focused on searching for dipeptidyl peptidase-4 inhibitors [56]. For the modelling, pIC_50_ = −log (IC_50_ (M)) was used. Finally, molecules were numbered according to Table S1 and Figure S1 in the Supporting Information. The set of 3D molecular structures was optimised at the molecular mechanics level using the universal force field (UFF) in the Avogadro program [57].

For the modelling, topological descriptors were used based on their good performance demonstrated in previous studies reported by our group [56,58,59,60]. The calculations were performed using the TOpological MOlecular COMputer Design suite (ToMoCoMD), which includes the programs QuBils MAS [58,61], allowing the calculation of 2D descriptors, and QuBils MIDAS [36], for the calculation of 3D descriptors, both based on algebraic calculations of linear, bilinear, and quadratic indices. A set of 945 descriptors was used for the modelling (865 2D and 80 3D). This set of descriptors was selected using Shannon’s entropy of 0.7 and Spearman’s coefficient of 0.7 as the cut-off points in a supervised manner with the pIC_50_ values as the response variable. Descriptors were eliminated by applying the Shannon entropy cut-off when more than 30% of the compounds had the same descriptor value, and the maximum allowable correlation between two descriptors was set using the Spearman coefficient cut-off. Other descriptors were obtained using the Drug Likeness Tool (DruLiTo), and electronic descriptors were calculated from the energy values of the HOMO and LUMO orbitals, which were obtained from the structure optimisation using the semiempirical PM3 method in Gaussian16 software [62]. Finally, the compounds in the dataset were divided into training and test sets of 75% and 25%, respectively, using Ward’s clustering method, which involves a hierarchical cluster analysis for a rational division of molecules [63] to minimise the sum of squares error in the groups [64].

### 3.2. Variable Selection

Feature selection and classification methods are essential for building robust predictive models, particularly in scenarios where the number of molecular descriptors exceeds the number of samples [65]. These techniques not only enhance model performance but also reduce the dimensionality of the feature space, preventing overfitting and improving interpretability [66,67]. In this study, feature selection was performed using evaluators such as WrapperSubsetEval, in combination with machine learning algorithms including GaussianProcesses, LinearRegression, SMOreg, IBK, and RandomForest. These methods were chosen for their ability to handle high-dimensional data and their effectiveness in identifying relevant features in chemical datasets. Feature classification involves selecting a subset of variables from the original set based on a specific criterion that prioritises the most relevant features for the predictive model. To implement these algorithms, we used Weka 3.8, a widely recognised tool for data analysis and its applicability in chemoinformatics [68].

### 3.3. Virtual Screening

Screening was performed using the obtained best model equation and applied to Food and Drug Administration (FDA)-approved drug data provided by DrugBank [29] and a natural products database obtained from Analyticon Discovery [38]. The pIC_50_ values were obtained for a total of 15,424 structures, and then an analysis of the applicability domain with respect to the training set was performed to select the best candidates, which were selected for further analysis based on molecular docking, molecular dynamics, and absorption, distribution, metabolism, and excretion test (ADME) [44].

### 3.4. Applicability Domain

The applicability domain (AD) analysis was performed using a set of descriptors obtained for the best model and applied to the internal database used for modelling and the external database consisting of FDA approved drug and natural product data. The AD analysis performs a structural similarity assessment and calculation of the similarity of a chemical to a set [69]. This step was performed using the methods Ranges, to deal with linearly dependent descriptors; Euclidean distance, the distance-based approach that evaluates similarities in descriptor space; city block distance and Probability from the AMBIT Discovery program [70]. The analysis of the applicability domain was based on a consensus score of the four applied methods. The score represents the proportion of the four methods that consider a molecule to be within the applicability domain, with scores ranging from zero to one indicating whether the molecule is outside or within the domain [71]. If three or more methods consider a compound to have a score ≤ 0.25, that molecule is excluded from further analysis [58].

### 3.5. Absorption, Distribution, Metabolism, and Excretion (ADME) Predictions

ADME parameters were calculated for both screening and internal datasets using the SwissADME software, which computes physicochemical descriptors and predicts ADME parameters, pharmacokinetic properties, drug-likeness, and compatibility with medicinal chemistry for single or multiple small molecules to support drug discovery. Among the internal methods, SwissADME employs tools such as the boiled egg diagram, iLOGP, and the Bioavailability Radar [38].

The boiled egg model developed by Daina [72], which predicts gastrointestinal absorption and brain penetration capacity. It is based on Egan’s egg model [73], which provides a visual representation to distinguish well-absorbed from poorly absorbed molecules based on their lipophilicity and polarity, typically described by the n-octanol/water partition coefficient. The model uses the log P method developed by Wildman and Crippen (WLOGP), which shares similarities with ALOGP98 but offers a more comprehensive chemical description [40]. Additionally, it calculates the topological polar surface area (tPSA), a widely accepted technique for estimating PSA using a 2D fragment-based approach [74]. The boiled egg model has proven to be highly practical for drug discovery and medicinal chemistry applications [44,71].

### 3.6. Molecular Docking

A docking study was performed on 54 molecules from the model database, using tyrosinase protein extracted from the Protein Data Bank (PDB) [75] with code 2Y9X [76]. The protein was pre-processed by removing water molecules, repairing the structure, and calculating charges. This was conducted using the AutoDock 4.2.6 program with AutodockTools [77], modifying the AutoGrid file to recognise the parameters for copper (Cu) and holmium (Ho) metal atoms in the protein structure and ligands.

The grid box was centred at coordinates −10.021, −28.823, and −43.596 (X, Y, and Z), as determined by the work of KaiTang and YiJiang [20]. The grid size was set to 40 points to ensure proper coverage of the ligands under study. Three docking simulations were performed, using kojic acid and the protein’s natural ligand as reference compounds.

### 3.7. Molecular Dynamics

Molecular dynamics (MD) simulations were conducted over a 200 ns trajectory using the GROMACS 2020.1 software package [78] for Linux-based environments. The pressure was coupled at 1 bar using the Parrinello–Rahman barostat, and the temperature was maintained at 300 K using the V-rescale thermostat. The simulations utilised the Amber99bsc1 force field [79], with the TIP3P water model [80] for solvation. The system was neutralised with sodium and chloride ions as indicated by the topology file and by utilising the GROMACS tool genion.

Protein and ligand preparations were tailored to the system’s requirements. The tyrosinase protein (PDB ID: 2Y9X) was pre-processed using Chimera [81] to remove water molecules, the natural ligand, and hydrogen atoms. Ligand topologies were generated using the Antichamber Python Parser Interface (ACPYPE 1.0) [82], which employed the general amber force field (GAFF) [79] for accurate representation of small organic molecules. The input files were processed with OpenBabel [83].

The simulation box was generated using gmx editconf, followed by solvation with the gmx solvate tool using a cubic box and the TIP3P water model. The system was then neutralised with gmx genion, where sodium and chloride ions were added to maintain neutrality.

Additionally, copper atoms were frozen in all three dimensions (X, Y, and Z) during the simulation using the freezegrps and freezedim options.

The system was initially equilibrated over 100 ps at 300 K using the Berendsen thermostat in two stages: first with constant volume (NVT ensemble), followed by the NPT ensemble where both pressure and temperature were controlled. Only bonds involving hydrogen atoms were constrained using the LINCS algorithm, with an accuracy of 1 iteration (lincs_iter = 1) and fourth-order expansion (lincs_order = 4).

Additional parameters included the following:

- A 2 fs time step (dt = 0.002 ps).
- The Verlet cutoff scheme for non-bonded interactions, with a short-range van der Waals cutoff of 1.2 nm and long-range electrostatics treated via particle mesh Ewald (PME) [84] with a 1.2 nm cutoff.
- Temperature coupling was performed in two groups (Protein_LIG and Water_and_ions) to ensure proper control over the system’s temperature, with a time constant (tau_t) of 0.1 ps for each group.
- The system was subjected to periodic boundary conditions (PBC) in all three dimensions, using pbc = xyz.
- SHAKE was not employed, as the LINCS algorithm suffices for constraints in this system. Dispersion corrections were not applied for proteins using the Amber99bsc1 force field [81].

The production MD run was initiated after the system was equilibrated in the NPT ensemble. The generated trajectory was analysed for various structural properties, including root mean square deviation (RMSD), root mean square fluctuation (RMSF), hydrogen bonding, binding energy, and radius of gyration.

For the binding energy calculations, interaction energy between the protein and ligand was extracted using gmx energy with a specific selection of the interaction terms. For RMSD and RMSF analysis, the trajectory was processed using gmx rms and gmx rmsf, respectively, with reference to the starting structure to monitor the stability of the protein-ligand complex.

### 3.8. Free Energy Calculations

Free energy calculations were performed in all the studied systems using the molecular mechanics Poisson–Boltzmann surface area (MM-PBSA) method. The binding free energy is obtained following Equation (2), which required the estimation of bonded and non-bonded interactions (EMM), polar (Gpolar), and non-polar (Gnonpolar) solvation energy calculated using the Poisson–Boltzmann equation and solvent accessible surface area (SASA), respectively.

G_X_ = E_MM_ + G_polar_ + G_nonpolar_ (2)

Each energy is calculated for the protein, the ligand, and the complex every 5 ns in the simulation interval of 10 to 200 ns.

**Figure 9:**
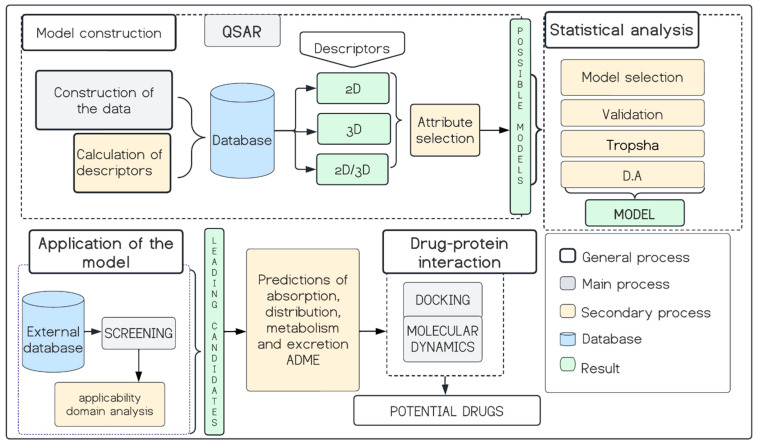
Workflow for the study of the inhibitory capacity of organic compounds on the enzyme tyrosinase by QSAR modelling, docking and molecular dynamics.

A schematic of the steps mentioned in this section is presented in Figure 9, as well as the methodology followed in the sections discussed in detail below.

## 4. Conclusions

Different machine learning algorithms were used for the modelling process, obtaining a group of models. Of these, the model with the best statistical parameters was Model 3M, obtained by MLR (Multiple Linear Regression), with a Q^2^_ext_ = 0.915, a Q^2^_LOO_ = 0.803, and similar RMSE values. In addition, this model met the validation criteria of the Tropsha test and showed 100% coverage in the analysis of the applicability domain for the external data, which allows us to affirm that it is a predictive, reliable, and robust model. Model 3, when applied to the screening database, yielded the pIC_50_ values of the molecules in these external databases, and as a result, a group of 15 molecules with tyrosinase enzyme inhibitory potential was proposed. These molecules were evaluated based on the criteria of high activity (pIC_50_ ≥ 7.82) and low lipophilicity (consensus log Po/w ≤ 3.51). Of these, five molecules were shown to be potential drug candidates. A thorough analysis of the protein-ligand interaction in the training and test sets, as well as in the selected compounds, was performed using molecular dynamics and molecular docking techniques. Collectively, five residues—H61, H85, H244, E256, and N260—emerged as critical for ligand binding and potential inhibition. Therefore, we propose these five structures for further in silico studies on their applicability to the human tyrosinase enzyme, as well as experimental studies aimed at developing pharmaceutical formulations for melanoma treatment.

## Figures and Tables

**Table 1:** Parameters of the four predictive models with the best validation results using QSARINS.

Model	R^2^	Q^2^_Loo_	Q^2^_LMO_	Q^2^_ext_	K_xx_	MAE_tr_	MAE_cv_	s	F
M3	0.8687	0.8030	0.7806	0.9151	0.2396	0.2424	0.3003	0.3562	37.4824
M4	0.855	0.7808	0.7568	0.9545	0.2361	0.2837	0.3508	0.3742	33.4248
M6	0.8235	0.7488	0.7291	0.9127	0.1943	0.2965	0.356	0.4069	32.6646
M9	0.8.762	0.7826	0.7486	0.7771	0.2607	0.2565	0.3379	0.3565	28.2981

**Table 2:** Validation parameters under the Tropsha criterion.

Criterion	Leave-One-Out Validation		External Validation	
	Result	Assessment	Result	Assessment
R^2^ > 0.6	0.8687	Pass	0.8687	Pass
R^2^_val_ > 0.5	0.8030	Pass	0.9151	Pass
(R^2^_val_ − R_0_^2^)/R^2^_val_ < 0.1	0.0008	Pass	0.0040	Pass
(R^2^_val_ − R_0′_^2^)/R^2^_val_ < 0.1	0.0423	Pass	0.0005	Pass
abs (R_0_^2^ − R_0′_^2^) < 0.1	0.0334	Pass	0.0032	Pass
0.85 < k < 1.15	1.0016	Pass	1.0057	Pass
0.85 < k′ < 1.15	0.9901	Pass	0.9896	Pass

**Table 3:** Name, identifiers, and pIC_50_ of the five best compounds obtained in the screening.

Cod	Name IUPAC	Commercial Name	DrugBank Access Number	CalculatedpIC_50_
1S	(4R)-4-(ethylamino)-2-(3-methoxypropyl)-1,1-dioxo-2H,3H,4H-1lambda6-thieno [3,2-e][1,2]thiazine-6-sulfonamide	Brinzolamide	DB01194	8357
2S	(2S,5R,6R)-6-[(6S)-6-(2-azaniumylacetamido)-6-carboxylatohexanamido]-3,3-dimethyl-7-oxo-4-thia-1-azabicyclo [3.2.0]heptane-2-carboxylate	-	DB03820	8193
3S	Dodecanoic acid	Lauric acid	DB03017	7953
4S	6-(cyclohexyl methoxy)-8-(propan-2-yl)-9H-purin-2-amine	-	DB08247	7942
5S	N-{[(2S)-1-[(3R)-3-amino-4-(3-chlorophenyl)butanoyl]pyrrolidin-2-yl]methyl}-3-methanesulfonylbenzamide	-	DB08429	7828

**Table 4:** Compounds that presented the highest pIC_50_ and favourable D.S., compounds 1S to 5S.

Cod	D.S. Average	pIC_50_ Pre.	H Bond	H-Bonding Distance (Å)
1S	−7.350	8.357	ASN 260	2.77
2S	−7.873	8.193	HIS 85	3.23
			CYS 83	3.09
			MET 280	2.21
3S	−4.577	7.953	ASN 260	1.98
4S	−7.043	7.942	HIS 85	2.41
5S	−8.950	7.828	VAL 283	3.34
			MET 280	2.35
			HIS 85	3.24

**Table 5:** Results of the molecular docking study, docking score in kcal/mol, for the 15 best compounds from the screening data predicted by the QSAR model and their pre-pIC_50_ values.

Number	Docking Score			D.S. Average	pIC_50_ Pre.
1S	−7.32	−7.09	−7.64	−7.350	8.357
2S	−8.06	−8.23	−7.33	−7.873	8.193
3S	−4.61	−4.68	−4.44	−4.577	7.953
4S	−7.05	−7.07	−7.01	−7.043	7.942
5S	−9.46	−8.46	−8.93	−8.950	7.828
6S	−8.82	−8.26	−8.69	−8.590	7.821
7S	−8.01	−8.06	−8.02	−8.030	7.816
8S	−7.3	−7.29	−7.29	−7.293	7.779
9S	−8.36	−8.39	−8.27	−8.340	7.727
10S	−6.54	−6.24	−6.62	−6.467	7.506
11S	−8.21	−8.19	−8.19	−8.197	7.525
12S	−4.93	−4.94	−4.53	−4.800	7.384
13S	−8.62	−7.76	−7.59	−7.990	7.222
14S	−7.08	−7.08	−7.08	−7.080	7.046
15S	−7.48	−6.5	−6.73	−6.903	7.039
TROPOLONA	−4.39	−4.39	−4.39	−4.390	-

**Table 6:** Van der Waals, electrostatic, SASA, and binding energy (BE) (in kcal/mol) of the studied compounds.

	Van der Waal Energy	Electrostatic Energy	SASA Energy	Binding Energy
1S	−127.101	13.975	−13.481	−37.541
2S	−123.600	−43.783	−14.183	−58.897
3S	−121.350	−3.193	−14.545	−81.305
4S	−100.239	−9.749	−11.871	−58.395
5S	−117.633	3.132	−13.651	−48.059
1	−134.124	−23.746	−15.918	−73.032
2	−97.265	−4.652	−11.665	−50.099
16	−66.685	−20.318	−7.604	−46.119
20	−132.890	−43.267	−13.689	−61.021
25	−142.256	−43.267	−13.689	−61.021
26	−100.899	−47.307	−13.259	−45.756
28	−39.580	−14.562	−5.562	−30.439
33	−46.112	−12.488	−5.957	−23.152
50	−103.203	−81.459	−11.914	−70.130

## Data Availability

The raw data supporting the conclusions of this article will be made available by the authors upon request.

## Supplementary Materials

The following supplementary information can be downloaded at https://www.mdpi.com/article/10.3390/ph18030418/s1: Table S1. Molecules used for the development of the QSAR model (internal data); Figure S1. Set of molecular structures used for the construction of the QSAR model; Table S2. Models obtained from the variable search and selection process using WEKA; Table S3. Topological descriptors of model 3 and correlation matrix of the descriptors; Table S4. Applicability domain evaluation of the 15 best-predicted compounds from external data; Table S5. Applicability domain assessment performed using four evaluation methods with AMBIT for model 3 of the dataset; Table S6. ADME properties of the 15 best-predicted compounds from external data; Figure S2. Interaction of tropolone with the amino acids of tyrosinase; Table S7. Types and interaction distances of tropolone with tyrosinase amino acids; Figure S3. Interactions and types of interactions between tyrosinase protein amino acids and the main compounds with the highest pIC_50_ values from the screening data; Figure S4. Protein–ligand interactions of the ligands evaluated from 1S to 5S, along with tropolone, in the tyrosinase active site. The two copper atoms, characteristic of the enzyme’s active site, are represented as brown spheres; Table S8. Results of the molecular docking study for the internal data; Figure S5. RMSD plots of the dataset complexes during the 200 ns MD simulation. Main interacting amino acids for the ligands in the modelling database; Figure S6. RMSD plots of the ligands in the dataset during the 200 ns MD simulation; Figure S7. RMSF plots of the ligands in the dataset during the 200 ns MD simulation; Figure S8. Main interaction zones from the molecular dynamics results; Figure S9. The main interactions of the screened ligands with their respective amino acids were evaluated over a 200 ns period during the molecular dynamics simulations.
