# Supplementary material for: Discovering New Tyrosinase Inhibitors by Using In Silico Modelling, Molecular Docking, and Molecular Dynamics
Source: Pharmaceuticals (Basel). 2025 Mar 16;18(3):418. doi: 10.3390/ph18030418 (PMC11946302; doi:10.3390/ph18030418)
Supplement: Supplementary file 1 [file pharmaceuticals-18-00418-s001.zip › pharmaceuticals-3388048-supplementary.pdf]

Discovering New Tyrosinase Inhibitors by Using In Silico Modelling, Molecular Docking, and Molecular Dynamics

Kevin A. OréMaldonado [1], Sebastián A. Cuesta [2,3], José R. Mora [2,*], Marcos A. Loroño [1,*] and José L. Paz [4]

[1] Departamento Académico de Química Fisicoquímica, Facultad de Química e Ingeniería Química, Universidad Nacional Mayor de San Marcos, Lima 15081, Peru
[2] Grupo de Química Computacional y Teórica (QCT-USFQ), Departamento de Ingeniería Química, Universidad San Francisco de Quito, Diego de Robles y Vía Interoceánica, Quito 170901, Ecuador
[3] Department of Chemistry, Manchester Institute of Biotechnology, The University of Manchester, Manchester M17DN, UK
[4] Departamento Académico de Química Inorgánica, Facultad de Química e Ingeniería Química, Universidad Nacional Mayor de San Marcos, Lima 15081, Peru;
* Correspondence: jrmora@usfq.edu.ec (J.R.M.); mloronog@unmsm.edu.pe (M.L.)

Supplementary material

Table S1. Molecules used for the development of the QSAR model (internal data).

Molecules	Status	IC_{50} st (uM)	pIC_{50}
1	Prediction	159.92	3.79609722
2	Training	441.22	3.35534481
3	Training	255.52	3.5925751
4	Training	115.14	3.93877377
5	Training	168.02	3.77463902
6	Training	198.42	3.70241455
7	Prediction	244.02	3.61257458
8	Training	50.12	4.29998894
9	Prediction	441.22	3.35534481
10	Training	149.62	3.82501035
11	Training	54.79	4.2612987
12	Training	156.12	3.80654146
13	Training	43.4	4.36251027
14	Training	44.58	4.35085994
15	Training	184.02	3.73513497
16	Training	198.62	3.70197702
17	Training	365.52	3.43708885
18	Training	595.82	3.22488492
19	Training	60.25	4.22004295

20	Prediction	32.41	4.48932097
21	Training	64.51	4.19037296
22	Training	126.42	3.89818421
23	Prediction	107.86	3.96713958
24	Training	31.34	4.50390101
25	Training	94.73	4.02351246
26	Training	117.32	3.93062795
27	Training	0.03	7.52287875
28	Prediction	0.07	7.15490196
29	Training	0.18	6.74472749
30	Training	1	6
31	Training	1.53	5.81530857
32	Prediction	1.95	5.70996539
33	Prediction	2.3	5.63827216
34	Training	2.79	5.5543958
35	Training	4.9	5.30980392
36	Training	450.72	3.34609317
37	Training	43.4	4.36251027
38	Training	42.54	4.37120251
39	Training	42.5	4.37161107
40	Prediction	42.73	4.36926711
41	Training	74.15	4.12988884
42	Training	301.38	3.52088557
43	Training	30.05	4.52215552
44	Training	105.45	3.97695342
45	Prediction	67.05	4.17360122
46	Training	191.85	3.7170382
47	Training	36.35	4.43949558
48	Training	38.65	4.4128505
49	Training	237.55	3.62424497
50	Prediction	275.55	3.55979958
51	Training	219.46	3.65864463
52	Prediction	291.85	3.5348403
53	Prediction	142.17	3.84719204
54	Training	110.12	3.9581338

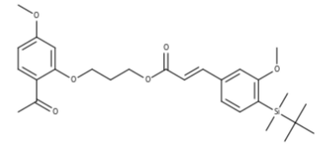
1

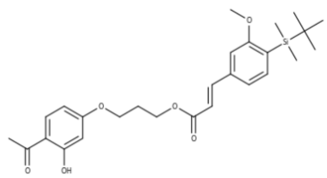
2

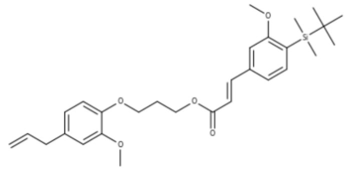
3

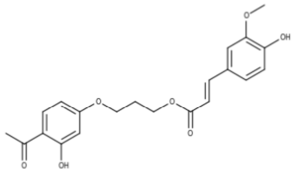
4

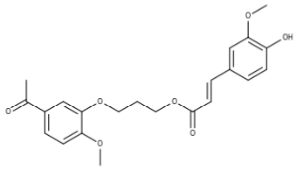
5

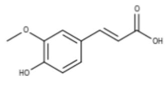
6

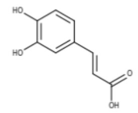
7

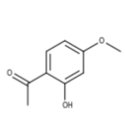
8

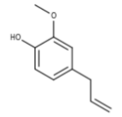
9

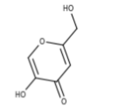
10

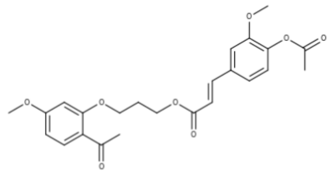
11

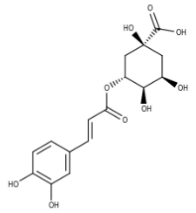
12

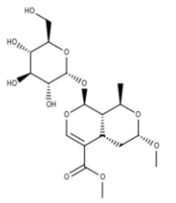
13

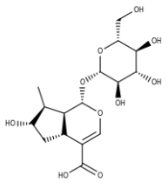
14

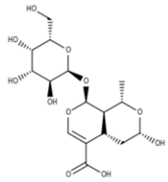
15

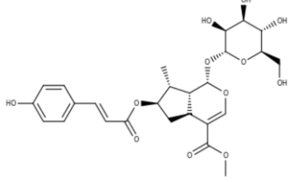
16

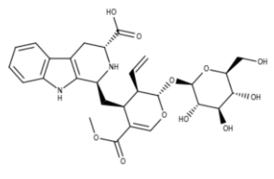
17

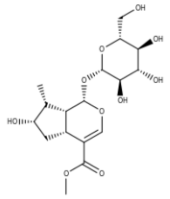
18

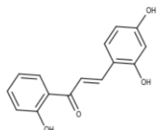
19

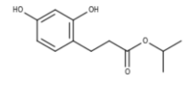
20

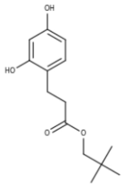
21

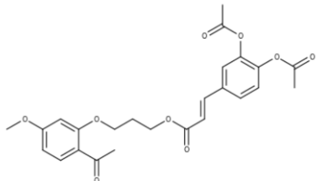
22

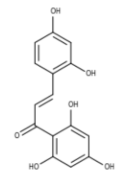
23

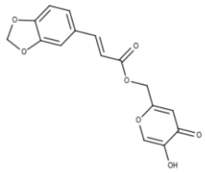
24

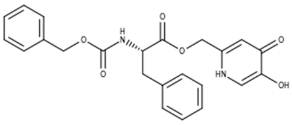
25

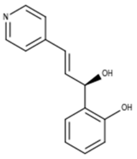
26

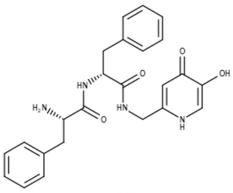
27

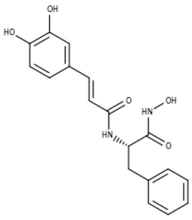
28

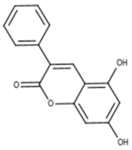
29

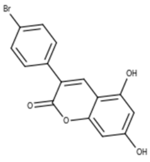
30

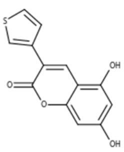
31

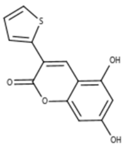
32

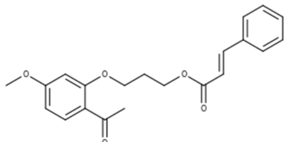
33

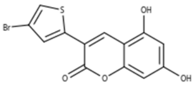
34

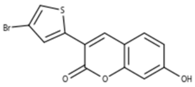
35

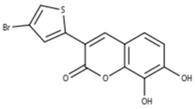
36

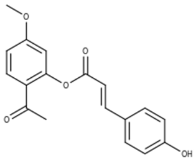
37

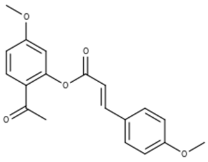
38

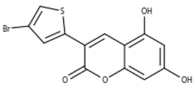
34

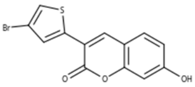
35

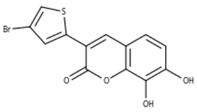
36

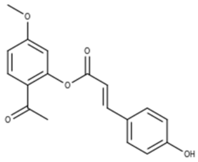
37

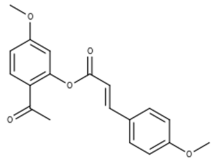
38

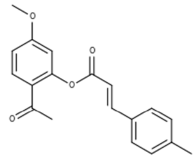
39

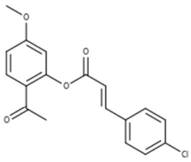
40

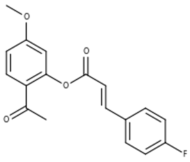
41

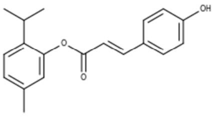
42

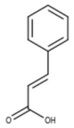
43

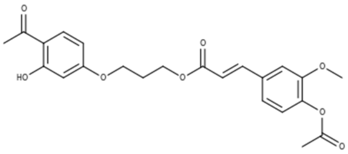
44

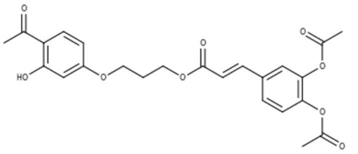
45

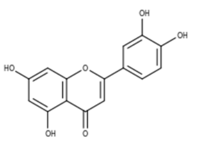

46

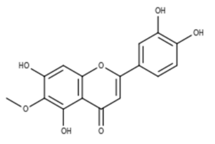

47

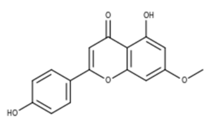

48

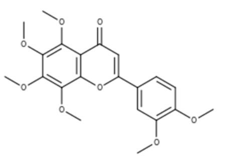

49

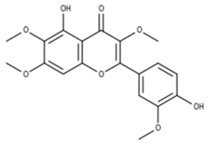

50

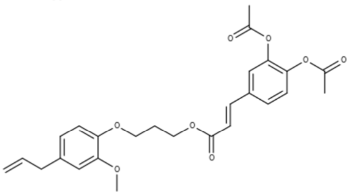

51

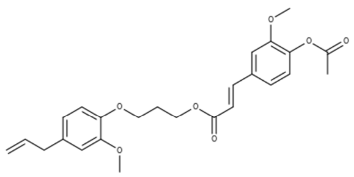

52

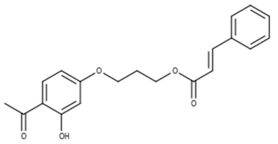

53

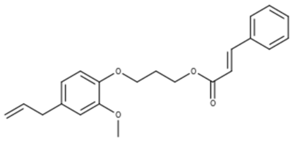

54

Figure S1. Set of molecular structures used for the construction of the QSAR model.

Table S2. Models obtained from the search and selection of variables using WEKA.

n°	Model	Number of descriptors	R	R^2
1M	1.12	5	6.049	0,36590401
2M	1.13	9	4.493	0,20187049
3M	1.15	7	8.337	0,69505569
4M	2.4	8	8.953	0,80156209
5M	2.5	10	9.182	0,84309124
6M	2.6	8	8.953	0,80156209
7M	2.10	5	8.109	0,65755881
8M	2.11	10	8.679	0,75325041
9M	2.12	5	8.109	0,65755881

10M	2.13	8	8.258	0,68194564
11M	2.14	8	8.258	0,68194564
12M	2.15	8	8.258	0,68194564
13M	3.12	8	6.927	0,47983329
14M	3.13	9	8.195	0,67158025
15M	3.15	9	8.195	0,67158025
16M	4.10	9	832	0,692224
17M	4.12	4	7.931	0,62900761
18M	4.13	8	8.331	0,69405561
19M	4.15	8	8.331	0,69405561
20M	6.12	4	7.931	0,62900761
21M	6.13	8	8.331	0,69405561
22M	6.15	8	8.331	0,69405561
23M	7.10	4	662	0,438244
24M	7.12	4	662	0,438244
25M	7.13	5	7.793	0,60730849
26M	7.15	6	757	0,573049
27M	8.10	8	8.224	0,67634176
28M	8.11	8	8.224	0,67634176
29M	8.12	3	7.801	0,60855601
30M	8.13	8	8.158	0,66552964
31M	8.14	8	8.158	0,66552964
32M	8.15	3	7.343	0,53919649
33M	9.10	4	662	0,438244
34M	9.12	4	662	0,438244
35M	9.13	5	7.793	0,60730849
36M	9.14	11	802	0,643204

37M	9.15	6	757	0,573049
38M	10.1	9	7.799	0,60824401
39M	10.4	8	7.882	0,62125924
40M	10.6	8	7.882	0,62125924
41M	10.7	4	6.534	0,42693156
42M	10.8	10	8.293	0,68773849
43M	10.9	4	6.534	0,42693156
44M	10.13	7	8.536	0,72863296
45M	10.15	7	8.536	0,72863296
46M	11.1	6	7.158	0,51236964
47M	11.2	6	7.158	0,51236964
48M	11.3	6	7.158	0,51236964
49M	11.4	2	6.427	0,41306329
50M	11.5	6	7.354	0,54081316
51M	11.6	2	6.427	0,41306329
52M	11.7	8	7.267	0,52809289
53M	11.8	8	7.267	0,52809289
54M	11.9	6	7.162	0,51294244
55M	11.10	5	7.738	0,59876644
56M	11.12	2	6.319	0,39929761
57M	11.13	3	7.129	0,50822641
58M	11.14	4	683	0,466489
59M	11.15	3	7.129	0,50822641
60M	12.1	4	6.034	0,36409156
61M	12.2	4	6.034	0,36409156
62M	12.3	4	6.034	0,36409156
63M	12.4	3	6.278	0,39413284

64M	12.5	3	6.278	0,39413284
65M	12.6	3	6.278	0,39413284
66M	12.7	3	4.851	0,23532201
67M	12.8	3	4.851	0,23532201
68M	12.9	1	-	0
69M	12.10	5	8.021	0,64336441
70M	12.11	5	8.021	0,64336441
71M	12.12	5	8.021	0,64336441
72M	12.13	5	7.342	0,53904964
73M	12.14	5	7.342	0,53904964
74M	12.15	2	7.481	0,55965361
75M	13.1	7	7.877	0,62047129
76M	13.2	7	7.877	0,62047129
77M	13.3	7	7.877	0,62047129
78M	13.4	5	7.767	0,60326289
79M	13.5	5	7.767	0,60326289
80M	13.6	5	7.767	0,60326289
81M	13.7	5	7.478	0,55920484
82M	13.8	5	7.478	0,55920484
83M	13.9	5	7.478	0,55920484
84M	13.10	2	833	0,693889
85M	13.11	6	8.472	0,71774784
86M	13.12	2	833	0,693889
87M	13.13	8	8.958	0,80245764
88M	13.14	8	8.958	0,80245764
89M	13.15	8	8.958	0,80245764
90M	14.12	7	7.805	0,60918025

91M	15.1	9	8.036	0,64577296
92M	15.2	9	8.036	0,64577296
93M	15.3	9	8.036	0,64577296
94M	15.4	7	7.869	0,61921161
95M	15.5	6	7.813	0,61042969
96M	15.6	7	7.869	0,61921161
97M	15.7	10	7.887	0,62204769
98M	15.8	8	7.746	0,60000516
99M	15.10	8	8.873	0,78730129
100M	15.11	8	8.814	0,77686596
101M	15.12	2	833	0,693889
102M	15.13	5	8.492	0,72114064
103M	15.14	9	8.887	0,78978769

Table S3. Topological descriptors of model 3 and correlation matrix of the descriptors.

Descriptor	Abbreviation	GV[3]	TS[6]	HM	TS[5]	GV[6	E
GV[3]_K_TrB_AB_nCi_3_M25(M15)_NS4_T_KA_r-s_MID	GV[3]	1					
TS[6]_K_TrC_AB_nCi_3_M27_SS1_T_KA_v_MID	TS[6]	-0,1428	1				
HM_B_AB_Ci(2.0;-2.0)_2_NS1_H_C_NSRW_r-v_MAS	HM	-0,2712	0,2103	1			

TS[5]_AM_TrF_AB_nCi_3_M25(M16)_SS7_T_KA_e_MID	TS[5]	-0,0829	0,001	-0,2462	1		
GV[6]_K_TrB_AB_nCi_3_M25(M8)_SS7_T_KA_p-s_MID	GV[6]	-0,0917	0,0468	0,0498	0,0337	1	
ES_AM_Tr_AB_nCi_3_M26(M1)_MP7_X_KA_psa-e-s_MID	ES	-0,2996	0,1356	0,206	0,1853	-0,222	1

Note:

KA: Means keep all elements in the matrix form (entire matrix)

AB: Atom-based Level

TrC: Threelinear-cubic Indices

TrF: Threelinear-linear Indices

3: Ternary

T: Total (Global) indices

nCi: Non-chiral indices

NS: Non Stochastic Matrix Order: 1

PSA: Topological Polar Surface Area

e: Electronegativity

s: Softness

M26: Full Summation sides (Wave-Edges Distance)

v: Vdw Volume

SS: Simple Stochastic Matrix Order: 1

Table S4. Evaluation of the applicability domain of the 15 best predicted compounds from the external data.

Database	Comp.	Calculated pIC50	in domain	Euclidean distance	in domain	City-block distance	in domain	Probability density	in domain	Consensus domain	in domain	Ranges	Drugbank_ID
FDA	1S	8,357	2	FALSE	1,029	TRUE	1,964	TRUE	0,00E+00	FALSE	0,5	TRUE	DB01194
	2S	8,193	2	FALSE	1,016	TRUE	1,898	TRUE	0,00E+00	FALSE	0,5	TRUE	DB03820
	3S	7,953	0	TRUE	1,008	TRUE	2,356	FALSE	3,47E-03	FALSE	0,5	TRUE	DB08231
	4S	7,942	3	FALSE	0,903	TRUE	1,976	TRUE	1,77E-04	FALSE	0,5	TRUE	DB08247

	5S	7,828	1	FALSE	1,045	TRUE	1,967	TRUE	0,00E+00	FALSE	0,5	TRUE	DB08429
	6S	7,821	2	FALSE	0,976	TRUE	1,902	TRUE	8,05E-04	FALSE	0,5	TRUE	DB13752
	7S	7,816	0	TRUE	0,949	TRUE	2,097	FALSE	1,13E-03	FALSE	0,5	TRUE	DB04797
	8S	7,779	2	FALSE	0,948	TRUE	1,929	TRUE	0,00E+00	FALSE	0,5	TRUE	DB06927
	9S	7,727	2	FALSE	0,933	TRUE	1,924	TRUE	1,00E-04	FALSE	0,5	TRUE	DB01333
	10S	7,506	0	TRUE	0,854	TRUE	1,826	TRUE	4,16E-03	FALSE	0,75	TRUE	DB00645
	11S	7,717	1	FALSE	0,882	TRUE	1,906	TRUE	7,10E-03	FALSE	0,5	TRUE	
	12S	7,717	1	FALSE	0,926	TRUE	1,928	TRUE	0,00E+00	FALSE	0,5	TRUE	
NP	13S	7,647	2	FALSE	1,032	TRUE	1,943	TRUE	0,00E+00	FALSE	0,5	TRUE	
	14S	7,642	2	FALSE	1,05	TRUE	1,908	TRUE	0,00E+00	FALSE	0,5	TRUE	
	15S	7,634	1	FALSE	0,864	TRUE	1,812	TRUE	0,00E+00	FALSE	0,5	TRUE	

Table S5. Application domain performed with 4 evaluation methods with AMBIT for model 3 of the data set.

Name	Observed pIC50	Ranges	in domain	Euclidean distance	in domain	City-block distance	in domain	Probability density	in domain	Consensus domain	in domain
1_test	3.796	0	TRUE	369	TRUE	640	TRUE	22.585	TRUE	1	TRUE
7_test	3.613	0	TRUE	220	TRUE	456	TRUE	3.556	TRUE	1	TRUE
9_test	3.355	1	FALSE	747	TRUE	1.507	TRUE	61	TRUE	0.75	TRUE
20_test	4.489	0	TRUE	509	TRUE	873	TRUE	25.754	TRUE	1	TRUE
23_test	3.967	0	TRUE	795	TRUE	1.358	TRUE	639	TRUE	1	TRUE
28_test	7.155	0	TRUE	835	TRUE	1.833	TRUE	55	TRUE	1	TRUE
32_test	5.710	0	TRUE	236	TRUE	502	TRUE	8.977	TRUE	1	TRUE
33_test	5.638	0	TRUE	426	TRUE	958	TRUE	1.872	TRUE	1	TRUE
40_test	4.369	0	TRUE	492	TRUE	1.163	TRUE	4.576	TRUE	1	TRUE
45_test	4.174	0	TRUE	399	TRUE	824	TRUE	3.813	TRUE	1	TRUE
50_test	3.560	1	FALSE	511	TRUE	1.007	TRUE	0	FALSE	0.5	TRUE
52_test	3.535	1	FALSE	515	TRUE	1.170	TRUE	137	TRUE	0.75	TRUE
53_test	3.847	0	TRUE	506	TRUE	1.002	TRUE	4.401	TRUE	1	TRUE

Table S6. ADME properties of the 15 best predicted compounds from the external data.

Molecule	Calculated pIC50	ESOL Class	Ali Class	Silicon-IT class	Consensus Log P o/ow
1S	8,357	VS	Soluble	Soluble	0.34
2S	8,193	HS	VS	Soluble	-2.39
3S	7,953	Soluble	MS	Soluble	3.51
4S	7,942	Soluble	MS	MS	2.84
5S	7,828	Soluble	MS	PS	2.73

6S	7,821	MS	MS	Soluble	3.26
7S	7,816	MS	MS	PS	3.69
8S	7,779	Soluble	MS	MS	2.61
9S	7,727	VS	Soluble	Soluble	0.51
10S	7,506	MS	MS	MS	3.69
11S	7,717	Soluble	MS	MS	3.57
12S	7,717	MS	MS	MS	4.14
13S	7,647	Soluble	MS	MS	3.16
14S	7,642	MS	MS	MS	4.18
15S	7,634	Soluble	Soluble	Soluble	2.13

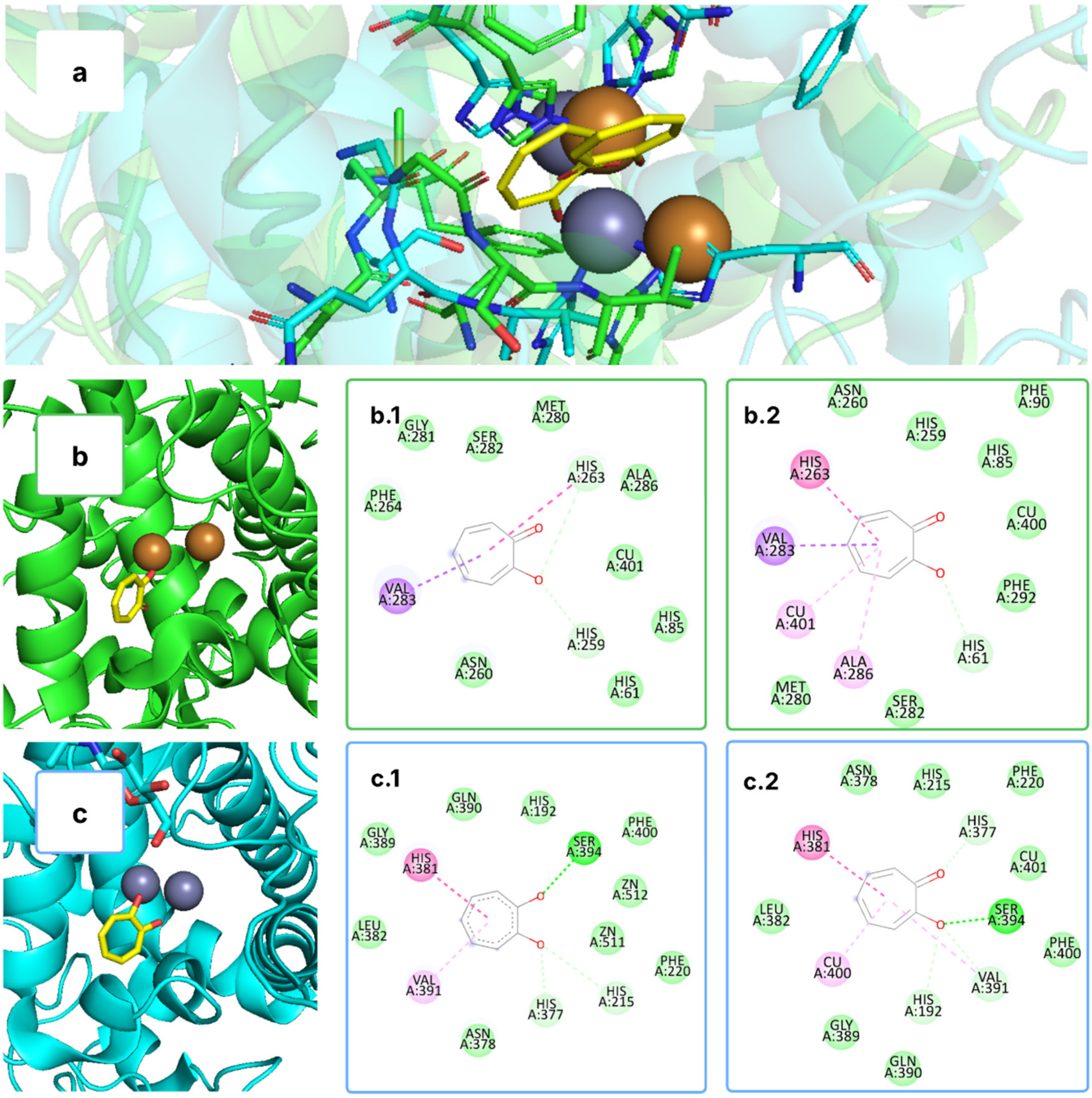

Figure S2. Comparison between the tropolone structure at the inhibition site of fungal and human tyrosinase: Panel (a) shows the general comparison of both structures. In panel (b.1), the main interactions between tropolone and the crystallized fungal enzyme are detailed, as reported in the PDB (b.10). Panel (b.2) illustrates the interactions of the docked tropolone during the redocking process for validation. Finally, in panel (c.1), the main interactions between tropolone and human tyrosinase, also reported in the PDB, are presented. Panel (c.2) shows the interactions of the docked tropolone during redocking and subsequent validation.

Table S7. Amino acids of interest in fungal and human enzymes as shown in Figure S2, and key interactions of tropolone with fungal tyrosinase.

<table>
<tr><th colspan="2">Fungal Tyrosinase</th><th colspan="2">Human Tyrosinase</th></tr>
<tr><th>b.1</th><th>b.2</th><th>c.1</th><th>c.2</th></tr>
<tr><td>VALINE</td><td>HISTIDINE</td><td>HISTIDINE</td><td>HISTIDINE</td></tr>
<tr><td>HISTIDINE</td><td>VALINE</td><td>VALINE</td><td>VALINE</td></tr>
<tr><td>SERINE</td><td>ALANINE</td><td>SERINE</td><td>SERINE</td></tr>
<tr><td>PHENYLALANINE</td><td>SERINE</td><td>ASPARAGINE</td><td>ASPARAGINE</td></tr>
<tr><td>ALANINE</td><td>ASPARAGINE</td><td>GLYCINE</td><td>GLYCINE</td></tr>
<tr><td>ASPARAGINE</td><td>PHENYLALANINE</td><td>LEUCINE</td><td>LEUCINE</td></tr>
<tr><td rowspan="8">Fungal Tyrosinase</td><td colspan="3">TROPOLONA (main interactions)</td></tr>
<tr><td>Interactions</td><td>Distances</td><td>A.A</td></tr>
<tr><td>Carbon hydrogen bond</td><td>3.79</td><td>His A 259</td></tr>
<tr><td>Pi donor hydrogen bond</td><td>3.91</td><td>His A 263</td></tr>
<tr><td>Pi sigma</td><td>3.42</td><td>Val A 283</td></tr>
<tr><td>Pi - pi stacked</td><td>05.07</td><td>His A 263</td></tr>
<tr><td>D.S</td><td colspan="2">-4.65</td></tr>
</table>

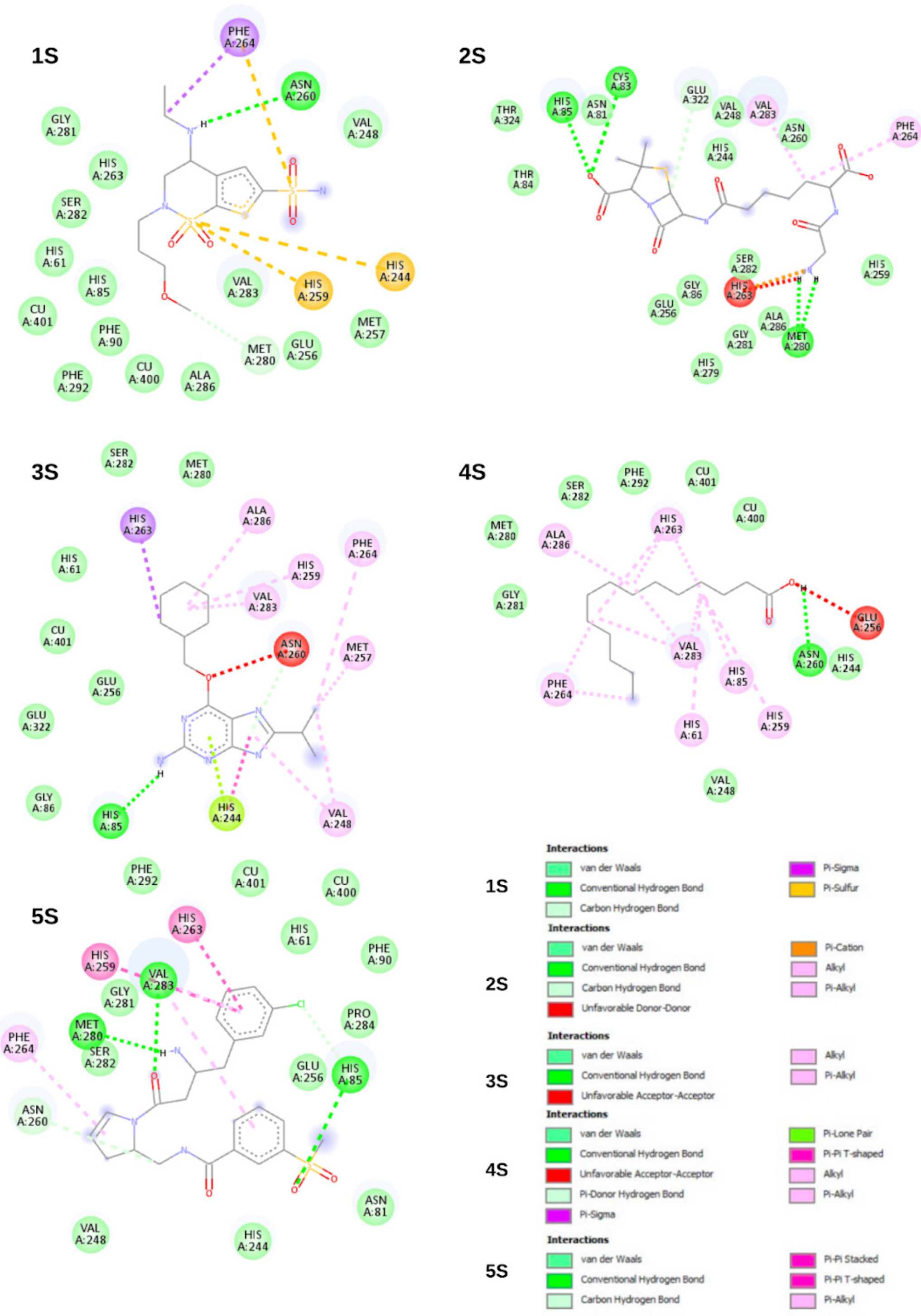

Figure S3. Interactions and types of interactions between protein tyrosinase amino acids and the major compounds with the highest pIC_{50} values from the screening data.

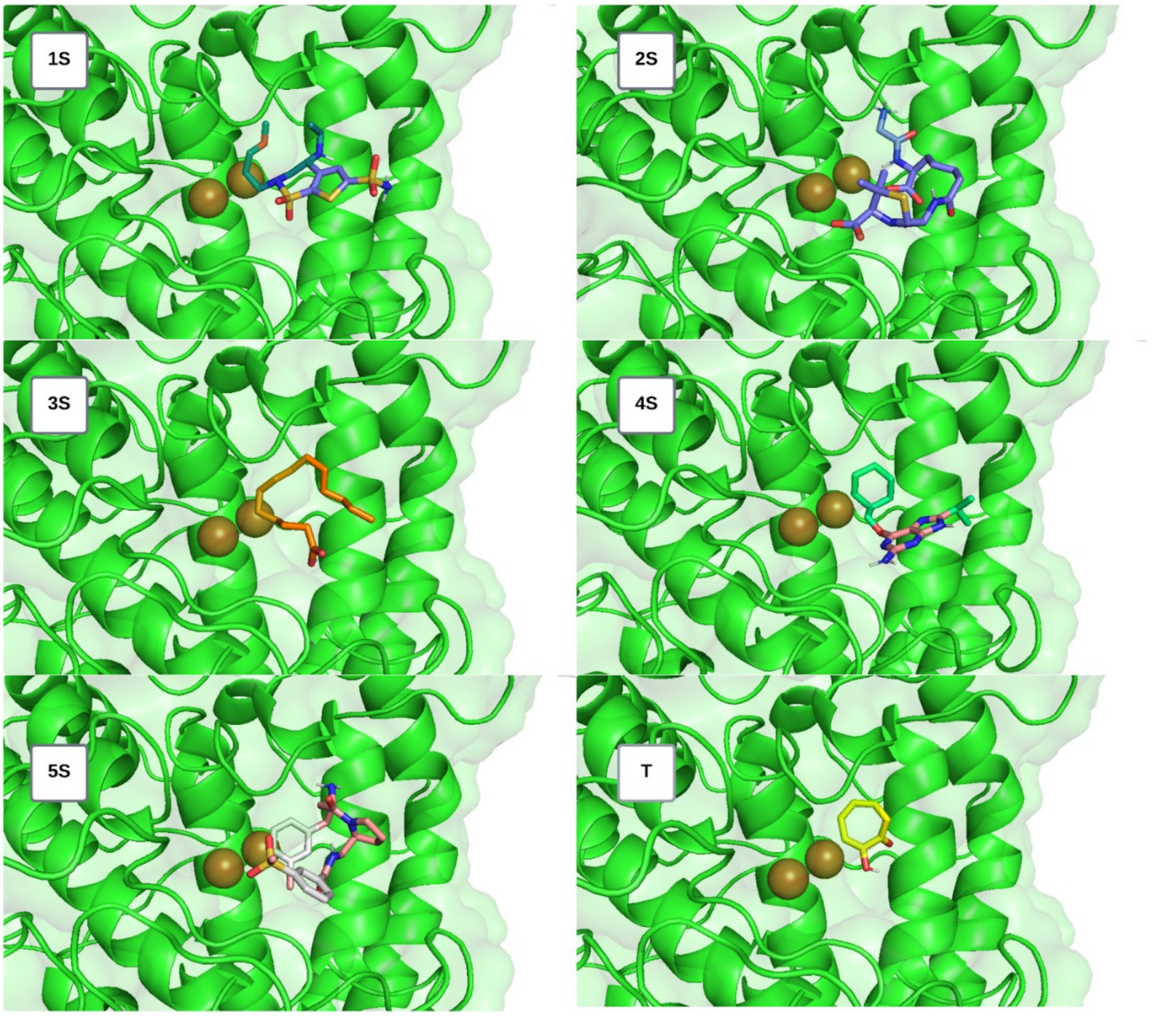

Figure S4. Protein-ligand interactions of the evaluated ligands from 1S to 5S, along with tropolone, at the active site of tyrosinase. The two copper atoms, characteristic of the enzyme's active site, are depicted as brown spheres.

Table S8. Results of the molecular docking study for the internal data.

Molecule	Binding energy			B.E promedio	pIC_{50}
1	-3.5	-4.51	-3.6	-3.8700	3.7961
2	-3.81	-3.32	-4.63	-3.9200	3.3553
3	-4.26	-4.26	-5.26	-4.5933	3.5926
4	-4.41	-4.37	-4.71	-4.4967	3.9388
5	-3.25	-3.83	-3.13	-3.4033	3.7746
6	-3.59	-4.02	-4.13	-3.9133	3.7024

7	-3.42	-3.21	-3.87	-3.5000	3.6126
8	-4.26	-4.06	-4.63	-4.3167	4.3000
9	-4.32	-4.17	-4.79	-4.4267	3.3553
10	-6.72	-7.81	-7.06	-7.2650	3.8250
11	-4.12	-4.34	-4.63	-4.3633	4.2613
12	-4.52	-3.69	-4.19	-4.1333	3.8065
13	-5.14	-5.04	-5.06	-5.0800	4.3625
14	-4.12	-3.75	-4.08	-3.9833	4.3509
15	-5.64	-5.82	-5.64	-5.7000	3.7351
16	-5.44	-5.43	-5.46	-5.4433	3.7020
17	-6.19	-6.19	-8.21	-6.8633	3.4371
18	-5.72	-5.65	-5.67	-5.6800	3.2249
19	-5.63	-5.84	-5.66	-5.7100	4.2200
20	-7.7	-7.56	-6.73	-7.3300	4.4893
21	-6.05	-6.39	-5.47	-5.9700	4.1904
22	-6.79	-6.61	-6.72	-6.7067	3.8982
23	-7.98	-7.13	-7.71	-7.6067	3.9671
24	-3.37	-4.14	-3.3	-3.6033	4.5039
25	-4.07	-4.82	-4.69	-4.5267	4.0235
26	-6.36	-6.65	-6.79	-6.6000	3.9306
27	-5.42	-5.78	-5.85	-5.6833	7.5229
28	-4.59	-4.38	-4.47	-4.4800	7.1549
29	-4.92	-5.12	-4.9	-4.9800	6.7447
30	-5.15	-5.26	-5.07	-5.1600	6.0000
31	-5.41	-5.71	-5.44	-5.5200	5.8153
32	-4.55	-4.26	-5.8	-4.8700	5.7100
33	-6.59	-6.57	-6.75	-6.6367	5.6383
34	-4.5	-4.13	-5.17	-4.3150	5.5544
35	-6.2	-5.99	-6.05	-6.0800	5.3098
36	-5.5	-5.39	-5.48	-5.4567	3.3461
37	-5.93	-5.92	-5.91	-5.9200	4.3625
38	-5.37	-5.38	-5.37	-5.3733	4.3712
39	-5.37	-5.36	-5.34	-5.3567	4.3716
40	-5.77	-5.74	-5.81	-5.7733	4.3693
41	-6.42	-6.43	-6.43	-6.4267	4.1299
42	-6.78	-6.76	-6.77	-6.7700	3.5209
43	-5.61	-5.27	-4.88	-5.2533	4.5222
44	-5.49	-5.18	-5.19	-5.2867	3.9770
45	-4.99	-5.27	-4.98	-5.0800	4.1736
46	-5.49	-5.68	-6.04	-5.7367	3.7170

47	-5.18	-5.48	-5.2	-5.2867	4.4395
48	-5.8	-5.86	-5.46	-5.7067	4.4129
49	-5.33	-5.25	-5.29	-5.2900	3.6242
50	-6.49	-6.55	-6.7	-6.5800	3.5598
51	-7.28	-7.19	-7.26	-7.2433	3.6586
52	-5.72	-5.79	-5.81	-5.7733	3.5348
53	-5.65	-5.68	-5.59	-5.6400	3.8472
54	-6.02	-6.15	-6.05	-6.0733	3.9581
Tropolone	-4.39	-4.39	-4.39	-4.3900	

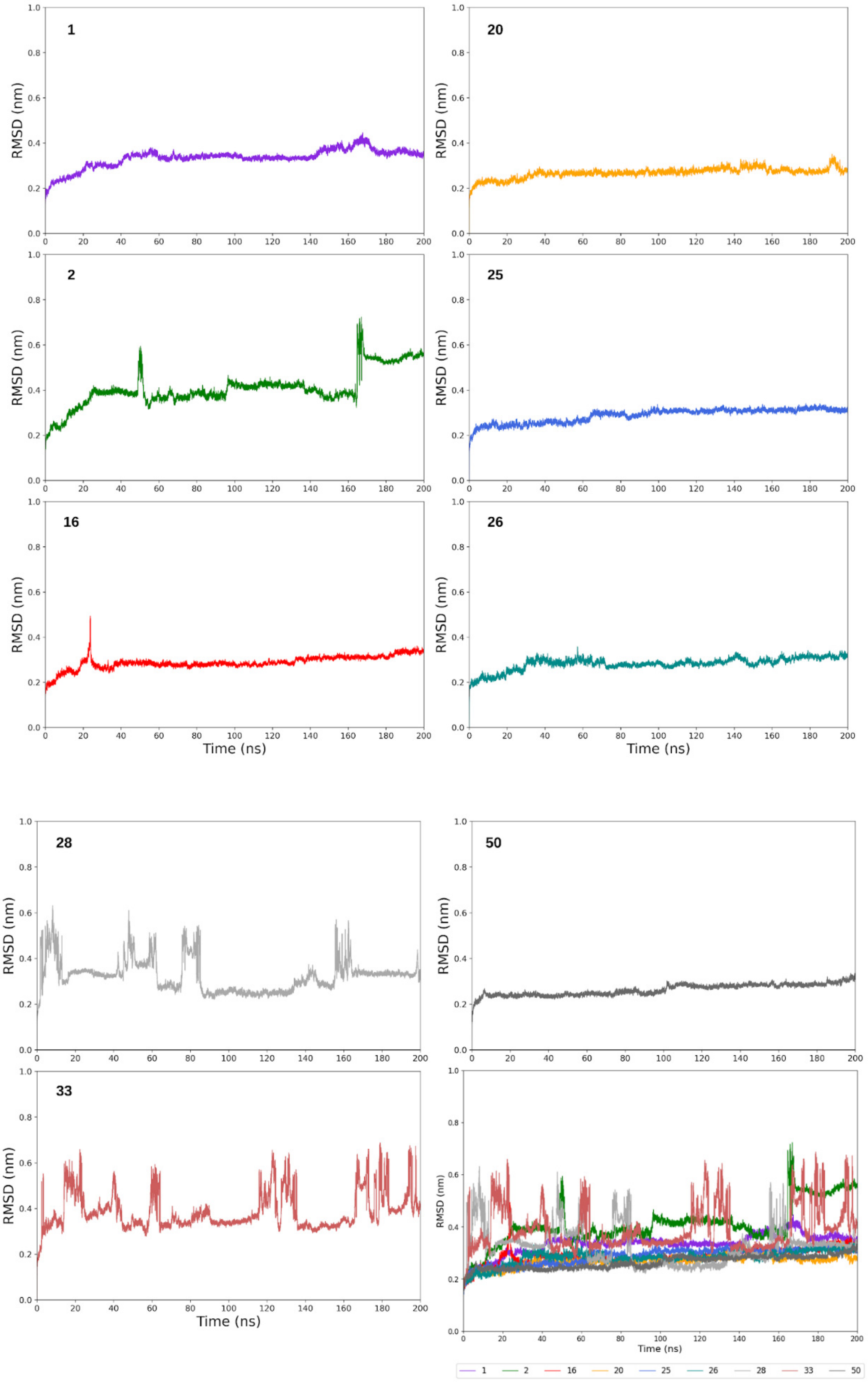

Figure S5. RMSD plots of the data set complexes during the 200 ns MD simulation. Main interacting amino acids for ligands in the modeling database

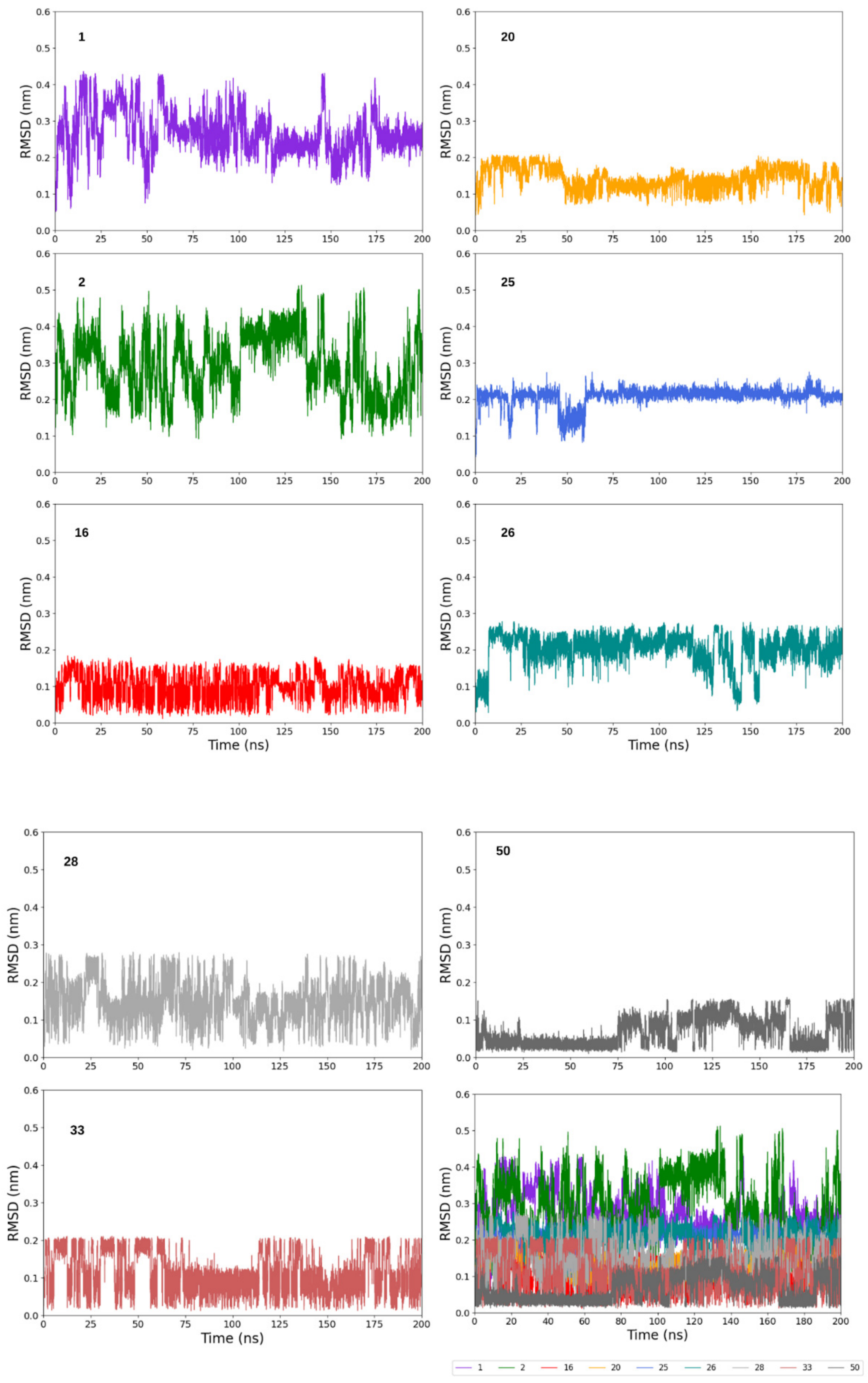

Figure S6. RMSD plots of the ligands in the data set, during the 200 ns MD simulation.

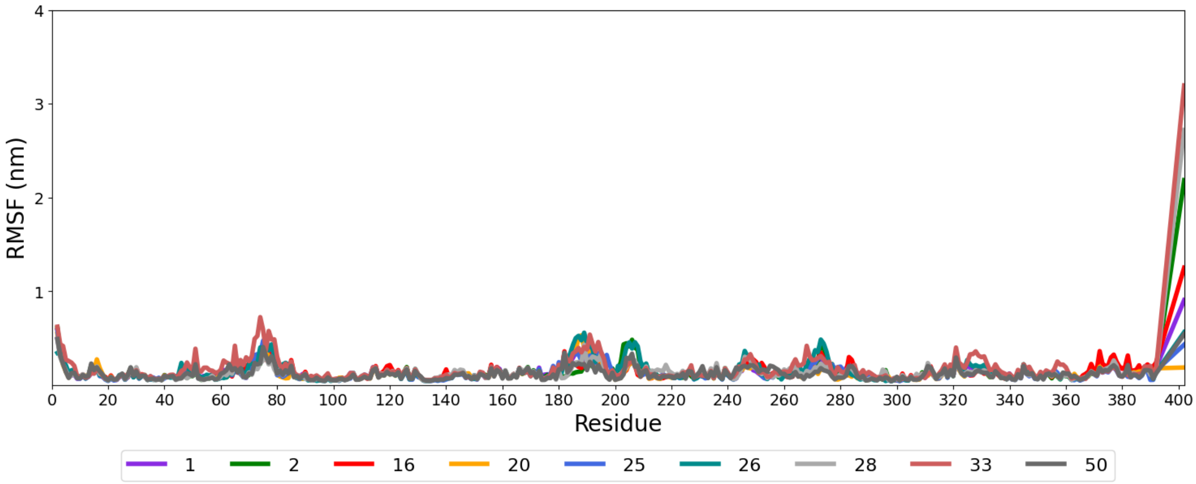

Figure S7. Plots of the RMSFs of the ligands in the data set during the 200 ns MD simulation.

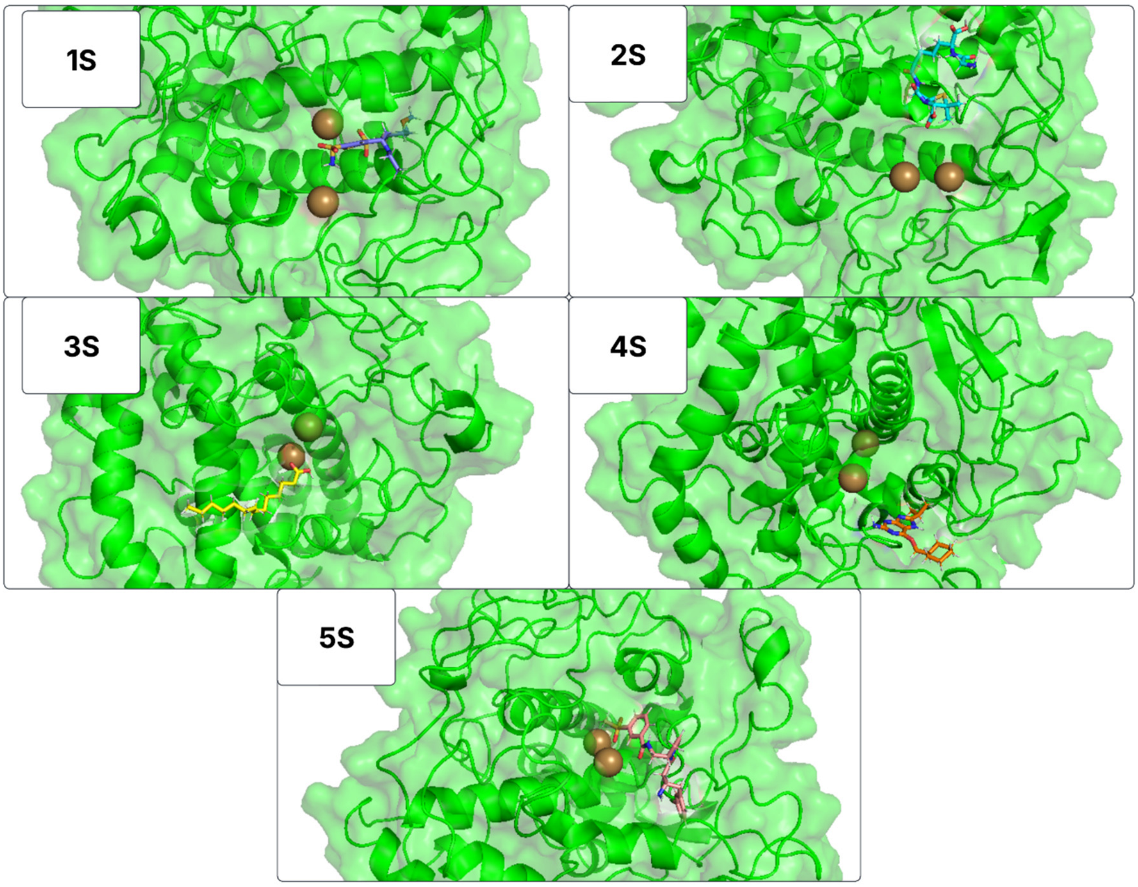

Figure S8. Main interaction zones from the molecular dynamics results.

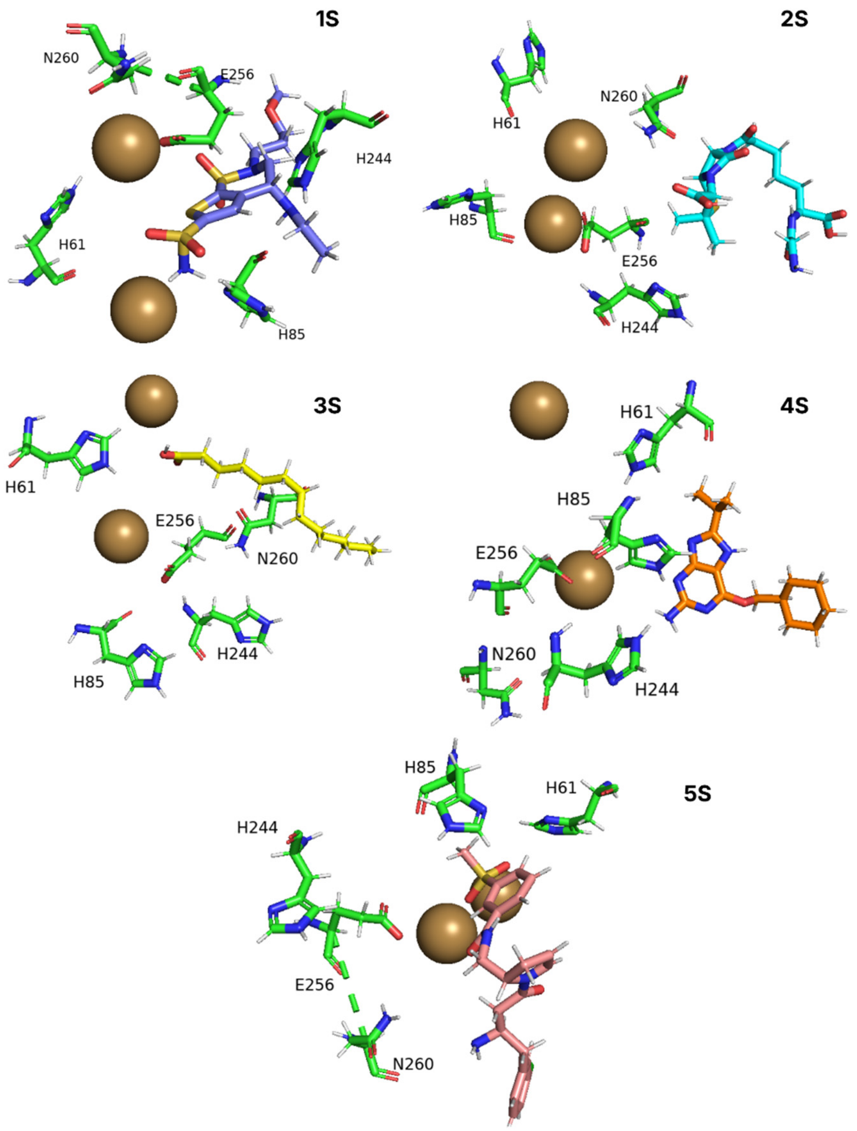

Figure S9. The main interactions of the studied screening ligands with their respective amino acids were evaluated over a period of 200 ns during molecular dynamics simulations
